# Genome‐wide CRISPR screen reveals an uncharacterized spliceosome regulator as new candidate immunotherapy target

**DOI:** 10.1002/imt2.70096

**Published:** 2025-11-28

**Authors:** Tong Shao, Chuanyang Liu, Jingyu Kuang, Sisi Xie, Ying Qu, Yingying Li, Lulu Zhang, Fangzhou Liu, Yanhua Qi, Tao Hou, Ming Li, Sujuan Zhang, Yu Liu, Zhixiang Yuan, Jiali Liu, Yanming Hu, Jingyang Wang, Chenghu Song, Shaowei Zhang, Lingyun Zhu, Jianzhong Shao, Aifu Lin, Wenjun Mao, Guangchuan Wang, Lvyun Zhu

**Affiliations:** ^1^ College of Science National University of Defense Technology Changsha China; ^2^ Key Laboratory of Multi‐Cell Systems, Shanghai Institute of Biochemistry and Cell Biology, Center for Excellence in Molecular Cell Science Chinese Academy of Sciences Shanghai China; ^3^ College of Life Science Zhejiang University Hangzhou China; ^4^ Department of Oncology, The Second Xiangya Hospital Central South University Changsha China; ^5^ Jiuquan Satellite Launch Centre Jiuquan China; ^6^ Department of Cardiothoracic Surgery, the Affiliated Wuxi People's Hospital of Nanjing Medical University, Wuxi People's Hospital, Wuxi Medical Center Nanjing Medical University Wuxi China; ^7^ Wuxi College of Clinical Medicine Nanjing Medical University Wuxi China

**Keywords:** antitumor immunity, C9ORF50, CRISPR screen, intrinsically disordered protein, liquid–liquid phase separation, RNA splicing, therapeutic targets

## Abstract

Cancer immune evasion is orchestrated by tumor‐intrinsic molecular constraints that remain incompletely defined. Here, we performed an in vivo genome‐wide clustered regularly interspaced short palindromic repeats (CRISPR) loss‐of‐function screen to catalogue gene regulatory determinants of immune evasion in cancer cells. We identify *C9ORF50* as a novel splicing regulator whose inhibition profoundly sensitizes cancer to immune surveillance. Integrated multi‐omics profiling reveals this intrinsically disordered protein exhibits liquid–liquid phase separation properties and forms nuclear condensates that colocalize with spliceosome components. Genetic ablation correlates with intron retention in multiple spliceosome components and cytoplasmic accumulation of double‐stranded RNA, which is associated with type I interferon activation and enhances chemokine‐mediated T cell recruitment. As a result, *C9ORF50* inhibition amplifies tumor cell immunogenicity, enhancing T cell infiltration in poorly infiltrated tumors. Clinically, elevated *C9ORF50* expression correlates with poor survival and diminished lymphoid infiltration across malignancies. Therapeutic targeting of *C9ORF50* using RNA interference enhances T cell infiltration and suppresses tumor growth. Our work identifies *C9ORF50* as a candidate therapeutic target that modulates RNA splicing and tumor immunity, suggesting splicing regulation as a potential strategy to enhance immunotherapy responses.

## Highlights

- An in vivo genome‐wide CRISPR screen reveals 15 tumor‐intrinsic immune evasion regulators, including an uncharacterized intrinsically disordered protein C9ORF50.
- Integrated multi‐omics establishes C9ORF50 driving liquid–liquid phase separation in the nucleus, facilitating membraneless nuclear compartments formation and dynamic spliceosome organization essential for RNA processing.
- *C9ORF50* inhibition represents a potential therapeutic approach targeting a tumor‐intrinsic genetic‐immune axis, which induces spliceosomal intron retention, generating immunogenic dsRNA that activates type I interferon responses and CD8⁺ T‐cell recruitment, turning cold tumors hot.
- Tumor‐predominant expression with non‐essential roles in normal mouse physiology suggests *C9ORF50* as a candidate target warranting further safety evaluation.

## Graphical abstract

This study identifies the uncharacterized gene *C9ORF50* as a novel regulator of immune evasion. Functioning as an intrinsically disordered protein, C9ORF50 drives liquid‐liquid phase separation to facilitate spliceosome assembly and maintain RNA splicing fidelity. Genetic ablation or therapeutic inhibition of *C9ORF50* disrupts this process, leading to widespread intron retention in pre‐mRNAs of core spliceosome components. These unprocessed introns form immunogenic double‐stranded RNA (dsRNA) that accumulates in the cytoplasm. This dsRNA is sensed by innate immune receptors (RIG‐I/MDA5), activating the TBK1‐IRF3 axis and subsequent type I interferon signaling. Consequently, this enhances the production of T‐cell chemoattractants (CXCL9/10/11), promoting robust CD8⁺ and CD4⁺ T cell infiltration into tumors. Together, these findings reveal that *C9ORF50* inhibition effectively converts immune‐suppressive tumors into T cell‐inflamed lesions, thereby enhancing immunogenicity and suppressing tumor growth.

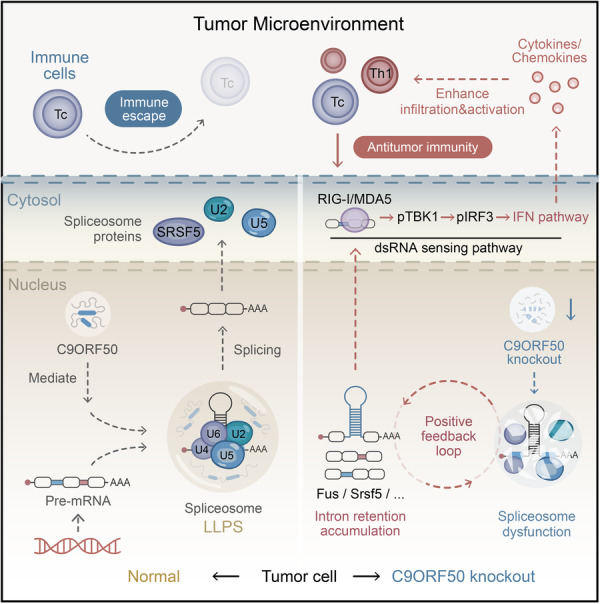

## INTRODUCTION

Cancer immunotherapies have significantly transformed the field of oncology [1, 2, 3, 4, 5, 6]. However, the achievement of sustained responses is still constrained by intrinsic tumor cell mechanisms that regulate immune evasion, metastasis, and dormancy [7, 8]. A crucial mechanism in this context is the suppression of “non‐self” immunogenicity, which facilitates immune escape and the establishment of an immune‐privileged tumor microenvironment (TME) characterized by T‐cell exclusion and diminished antitumor responses [2, 9, 10, 11, 12, 13]. Consequently, the identification of druggable regulators that can enhance tumor immunogenicity constitutes a vital area of research for improving immune‐mediated tumor eradication. High‐throughput CRISPR screening has accelerated the discovery of cell‐intrinsic vulnerabilities, revealing key targets such as the synthetic lethality target *WRN* in microsatellite instability‐high tumors, the myo‐inositol transporter *SLC5A3* in acute myeloid leukemia, apoptosis‐resistance drivers *MCL1* and *B3GNT2* identified through CRISPR activation screen, and the multidrug‐resistance regulator *PLK4* [14, 15, 16, 17]. These findings enable direct targeting of previously undruggable cancer mechanisms.

Within targetable tumor‐intrinsic processes, dysregulated RNA splicing emerges as a critical axis that not only drives oncogenesis and immune evasion but also contains pharmacologically exploitable nodes. As a well‐recognized cancer hallmark, aberrant splicing drives tumorigenesis and therapy resistance while providing diagnostic biomarkers and actionable targets [18, 19]. Key splicing regulators, including SRSF6, SF3A2, and SF3B1 critically promote malignant progression, motivating intensive pharmacological targeting of spliceosomal machinery [20, 21, 22, 23]. Clinically investigated agents employ distinct strategies: SF3B1 inhibitors alter survival‐associated transcripts through branchpoint recognition disruption [24, 25, 26]; herboxidiene impairs immune‐invasive pathways by blocking tri‐snRNP assembly [27]; while degraders like E7820 eliminate *RBM39* in metastatic tumors via *CUL4‐DCAF15* ubiquitin ligase hijacking [28]. Notably, emerging studies reveal synergistic potential between splicing modulation and immunotherapy. For example, RBM39 degradation or arginine methyltransferase inhibition has been shown to enhance tumor antigen presentation, which may counteract immune checkpoint blockade resistance [28, 29]. However, clinical translation faces significant challenges due to spliceosomal complexity, tumor heterogeneity, and on‐target toxicity concerns.

In this study, through an in vivo genome‐wide CRISPR screen in syngeneic colorectal models, we have identified *C9orf50*, a gene previously lacking characterization, as a novel target for cancer immunotherapy. C9ORF50 is structurally categorized as an intrinsically disordered protein (IDP) and plays a crucial role in regulating RNA splicing through liquid–liquid phase separation (LLPS). The genetic knockout of *C9orf50* induces an intrinsic innate immune response in cancer cells by disrupting splicing, thereby transforming immunologically cold tumors into inflamed phenotypes characterized by increased T cell infiltration and enhanced antitumor activity. The predominant expression of *C9ORF50* in tumors, along with its non‐essential role in normal physiological processes, identifies it as an optimal therapeutic target with minimal off‐target effects. Our research identifies *C9ORF50* as a candidate modulator of splicing fidelity that influences immune surveillance, thereby suggesting splicing regulation as a potential strategy to enhance immunotherapy.

## RESULTS

### Genome‐scale in vivo CRISPR screen identifies critical drivers of cancer progression

**Figure 1:**
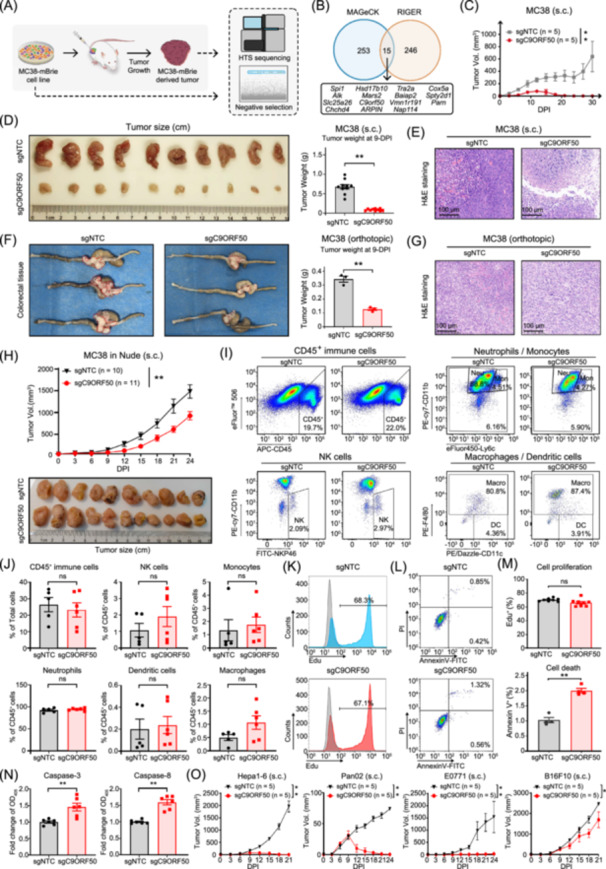
Genome‐scale *in vivo* clustered regularly interspaced short palindromic repeats (CRISPR) screen identifies *C9ORF50* as a critical driver of cancer progression. (A) Schematics of the experimental design of CRISPR knockout screen. (B) Venn diagram of the overlap of hits identified using RNAi Gene Enrichment Ranking (RIGER) analysis and Model‐based Analysis of Genome‐wide CRISPR/Cas9 Knockout (MAGeCK) analysis, revealing 15 genes significant regardless of criteria. (C) Tumor growth curve of subcutaneously transplanted MC38 cells transduced with *C9orf50*‐knockout sgRNA (sgC9ORF50, 5 mice/group) or NTC sgRNA (sgNTC, 5 mice/group) in C57BL/6 mice. s.c., subcutaneous. **p < 0.01 by two‐way ANOVA with Holm–Sidak's multiple comparisons test. Data are shown as mean ± SEM. (D) Representative photographs and weights of tumors from heterotopic tumor transplant. s.c., subcutaneous. **p < 0.01 by Student's *t* test. Data are shown as mean ± SEM. sgC9ORF50, *n* = 10 mice; sgNTC, *n* = 10. (E) Representative H&E images of tumors from (D). Scale bar, 100 μm. Images representative of 10 biological repeats. (F) Representative photographs and weights of tumors with orthotopic tumor transplant. Tumors were circled out by dashed lines. **p < 0.01 by Student's *t* test. Data are shown as mean ± SEM. sgC9ORF50, *n* = 3 mice; sgNTC, *n* = 3. (G) Representative H&E images of tumors in (D). Scale bar, 100 μm. Images representative of three biological repeats. (H) Tumor growth curve of nude mice transplanted with *C9orf50*‐deletion‐driven tumor cells (sgC9ORF50, *n* = 11 mice) or NTC tumor cells (sgNTC, *n* = 10 mice). Photographed tumors of nude mice transplanted with *C9orf50*‐deletion‐driven tumor cells. **p < 0.01 by two‐way ANOVA with Holm–Sidak's multiple comparisons test. (I) Leukocytes (CD45^+^ cells), neutrophils, monocytes, macrophages, DCs and NK cells infiltration in the tumor microenvironment of nude mice transplanted with *C9orf50*‐deletion‐driven tumor cells (sgC9ORF50, *n* = 6 mice) or NTC tumor cells (sgNTC, *n* = 5 mice) by flow cytometry analysis. Neu, Neutrophils; Mon, monocytes; Macro, macrophages. (J) Quantification of CD45^+^ cells, neutrophils, monocytes, macrophages, DCs and NK cells of (I). No significant difference (n.s.) by Student's *t* test. Data are shown as mean ± SEM, *n* = 6. (K) Flow cytometry shown the cell proliferation rate of MC38‐sgNTC or MC38‐sgC9ORF50 cells in vitro. (L) Cell apoptosis was analyzed by double staining with PI and Annexin V and detected by flow cytometry in vitro. (M) Statistical bar chart showing the cell proliferation and apoptosis rate of MC38‐sgNTC or MC38‐sgC9ORF50 cells. No significant difference (n.s.) *p* > 0.05, **p < 0.01 by Student's *t* test. Data are shown as mean ± SEM, *n* = 9 for cell proliferation assay, *n* = 3 for cell apoptosis assay. (N) Caspase‐3 and −8 activities from MC38‐sgNTC or MC38‐sgC9ORF50 cells. **p < 0.01 by Student's *t* test. Data are shown as mean ± SEM, *n* = 6. (O) Tumor growth curve of subcutaneously transplanted Hepa1‐6, Pan02, E0771, and B16F10 cells transduced with *C9orf50*‐knockout sgRNA (sgC9ORF50) or NTC sgRNA (sgNTC) in C57BL/6 mice. 5 mice/group. s.c., subcutaneous. *p < 0.05, **p < 0.01 by two‐way ANOVA with Holm–Sidak's multiple comparisons test. Data are shown as mean ± SEM.

To identify key genes involved in cancer progression under immune surveillance, we performed a genome‐wide in vivo CRISPR knockout screen in immunocompetent C57BL/6 mice. MC38 cells stably expressing Cas9 (MC38‐Cas9) were transduced with the mBrie genome‐wide CRISPR knockout sgRNA library (MC38‐Cas9‐mBrie) or a nontargeting sgRNA control (MC38‐Cas9‐NTC) (Figure 1A). While MC38‐Cas9‐mBrie and MC38‐Cas9‐NTC control tumors exhibited comparable growth through day 9, MC38‐Cas9‐mBrie tumors regressed from Day 9 to Day 20, suggesting that the loss of specific genes lead to enhanced immune rejection (*p* < 0.001; Figure S1A,B). To identify specific genes responsible for tumor rejection, we harvested MC38‐Cas9‐mBrie tumors at day 9 postinoculation, extracted genomic DNA, and performed second‐generation sequencing to quantify the changes of sgRNA abundance. Parallel sequencing of pre‐injection MC38‐Cas9‐mBrie cells and the original plasmid library revealed high coverage and strong interlibrary correlation (Figure S1C,D). The consistent sgRNA distribution across replicates and distinct profiles between tumor and cell libraries demonstrated robust screen performance and significant in vivo selective pressure (Figure S1E,F and Table S1). Using RNAi Gene Enrichment Ranking (RIGER) and Model‐based Analysis of Genome‐wide CRISPR/Cas9 Knockout (MAGeCK) algorithms for sgRNA enrichment analysis, we identified 261 and 268 significantly depleted genes, respectively (Figure S1G,H and Tables S2, 3). Convergent analysis yielded 15 high‐confidence candidates associated with tumor rejection, including previously established immunotherapy targets (*Alk, Spi1*), cytoskeleton regulators (*Baiap2, 2610034B18Rik/Arpin*), mitochondrial components (*Chchd4, Cox5a, Hsd17b10, Slc25a26, Mars2*), histone chaperones (*Nap1l4, Spty2d1*), RNA processing factors (*Parn, Tra2a*), and uncharacterized genes (*1700001O22Rik/C9orf50, Vmn1r191*) (Figure 1B, Figure S1H) [30, 31]. sgRNA read analysis confirmed marked depletion of all 15 candidates in tumors, surpassing a stringent FDR threshold of 0.2%. A direct comparison of sgRNA reads revealed marked depletion of sgRNAs targeting these 15 genes in tumors, with at least one sgRNA surpassing a stringent false discovery rate (FDR) threshold of 0.2% (Figure S1I–K). To further validate these findings, we selected one or two representative genes from each functional category for experimental validation and individually knocked out eight candidate genes: *C9orf50*, *Baiap2*, *Cox5a*, *Slc25a26*, *Spty2d1*, *Tra2a*, *Parn*, and *Nap1l4*. T7E1 assays confirmed efficient gene knockout (Figure S2A), and knockout lines demonstrated consistent tumor suppression in immunocompetent C57BL/6 mice compared to NTC controls (Figure S2B). Notably, knockout of known tumor suppressors *SetD2* and *Smad4* maintained normal tumor growth, confirming the specificity of our approach (Figure S2C). Collectively, this in vivo screen robustly identified high‐confidence candidate genes critical for tumor growth under immune pressure.

Given the limited understanding of the uncharacterized gene *C9orf50* identified in our screen, we sought to investigate its role in tumor growth and immune surveillance. In a heterotopic model, *C9orf50* knockout MC38 cells were transplanted subcutaneously into mice alongside NTC controls. Tumor growth curves revealed that *C9orf50* knockout MC38 cells formed significantly smaller tumors in immunocompetent C57BL/6 mice compared to NTC controls (Figure 1C). On day 9‐post‐transplantation, tumors were excised for direct measurement, revealing that *C9orf50*‐knockout tumors were notably smaller (Figure 1D, Figure S3A,B). Histological analysis showed a lower cell density in *C9orf50*‐knockout tumors (Figure 1E, Figure S3C). Similarly, in orthotopic colorectal tumor models, *C9orf50* knockout significantly reduced tumor growth compared to the NTC controls (Figure 1F,G, Figure S3D). Moreover, using a genetically engineered *C9orf50*‐knockout mouse model, we directly demonstrate that *C9orf50* deficiency protects against AOM/DSS‐induced colorectal tumorigenesis (Figure S3E,F). These results indicate that *C9orf50* knockout effectively impedes tumor progression in vivo.

To investigate the role of *C9orf50* knockout in the antitumor effects, we inoculated *C9orf50* knockout and NTC control MC38 cells into T cell‐deficient nude mice. *C9orf50* knockout only resulted in a modest reduction in tumor growth compared to controls (Figure 1H). Flow cytometric analysis revealed no significant differences in the overall infiltration of CD45^+^ immune cells, as well as the percentages of different innate immune cell subsets, including neutrophils, monocytes, dendritic cells, macrophages, and NK cells within CD45^+^ immune cells, between the two groups (Figure 1I,J, Figure S3G). Further analysis demonstrated that *C9orf50* knockout does not significantly affect tumor cell proliferation but induces apoptosis both in vivo and in vitro (Figure 1K–M, Figure S3H–J). This proapoptotic effect, primarily driven by activation of the extrinsic apoptosis mediator's caspase‐3 and caspase‐8 (Figure 1L–N), likely underlies the modestly reduced tumor growth observed in nude mice. Notably, this apoptotic mechanism aligns with prior findings that spliceosome inhibition could trigger extrinsic apoptosis via antiviral dsRNA‐sensing pathways [32]. Collectively, these findings suggest *C9orf50* deficiency primarily drives tumor regression via T cell‐dependent immunity, with a subsidiary contribution from extrinsic apoptosis in tumor cells.

To further investigate whether *C9orf50* knockout affects the growth of other cancer types, we knocked out *C9orf50* in Hepa1‐6 (Hepatoma), Pan02 (pancreatic cancer), E0771 (triple‐negative breast cancer), and B16F10 (melanoma), and transplanted these cells into C57BL/6 mice (Figure S3K). Consistent with our observations in the MC38 model, *C9orf50* knockout in these cells significantly inhibited tumor growth and resulted in better survival compared to NTC controls (Figure 1O, S3L). Collectively, these findings suggest that *C9orf50* knockout enhances tumor rejection in various cancer types, particularly those of gastrointestinal origin.

### C9ORF50 is a canonical intrinsically disordered protein driving LLPS

**Figure 2:**
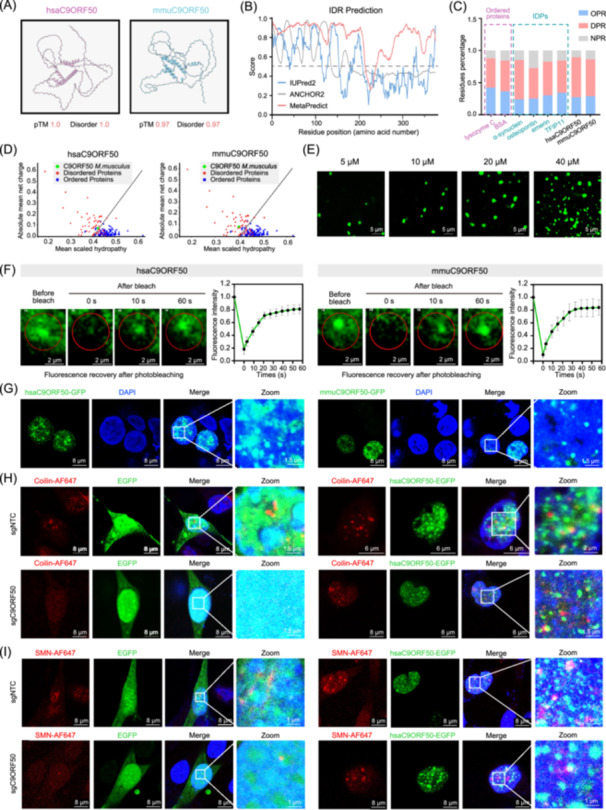
C9ORF50 drives liquid–liquid phase separation (LLPS) to form dynamic nuclear condensates. (A) Three‐dimensional (3‐D) structures of the C9ORF50 orthologue genes in human (hsaC9ORF50) and mouse (mmuC9ORF50) were predicted using AlphaFold3. (B) Intrinsic Unstructured Protein Disorder Predictor 2 (IUPred2), ANCHOR2 and MetaPredict analysis the disordered regions (IDRs) in C9ORF50. (C) Occurrence of OPR (order‐promoting residues), DPR (disorder‐promoting residues) and NPR (non‐promoting disorder/order residues) in the sequence of well‐ordered proteins (lysozyme C, BSA), known IDPs (Osteopontin, α‐Synuclein, Emerin), and C9ORF50. (D) Charge‐Hydropathy plot of the hsaC9ORF50 and mmuC9ORF50 using the PONDR algorithm. The solid black line represents the border between ordered and disordered phases. (E) C9ORF50 fused into liquid droplets in a concentration‐dependent manner in vitro. Scale bar, 5 μm. Images representative of three biological repeats. (F) The fluorescence intensity of husC9ORF50‐GFP (left) and mmuC9ORF50‐GFP (right) droplets recovered after bleaching during fluorescence recovery after photobleaching (FRAP) assay. Quantification of fluorescence intensity recovery in the bleached region of C9ORF50 droplets. Time 0 indicates the photobleaching pulse. Scale bar, 2 μm. Error bars, SEM of three independent experiments. (G) Subcellular localization of husC9ORF50‐GFP and mmuC9ORF50‐GFP in MC38 cells. Scale bar, 8 μm. Images representative of three biological repeats. Representative immunofluorescence of Coilin (H) and survival of motor neurons protein (SMN) (I) in MC38‐sgNTC and MC38‐sgC9ORF50 cells under four conditions: upper left, co‐localization of GFP and Coilin in MC38‐sgNTC cells; upper right, co‐localization of C9ORF50‐GFP and Coilin in MC38‐sgNTC cells; lower left, co‐localization of GFP and Coilin in MC38‐sgC9ORF50 cells; lower right, co‐localization of C9ORF50‐GFP and Coilin in MC38‐sgC9ORF50 cells. Column 4 displays 5× zoom of boxed regions of Merge. Scale bar, 6 μm, 8 μm, 1.5 μm, and 1 μm as indicated. Images representative of 3 biological repeats.

Structural analysis revealed that human and mouse C9ORF50 harbor a conserved domain of unknown function (DUF4685) but lack resolvable tertiary architecture (Figure 2A, Figure S4). Computational disorder profiling using IUPred2, ANCHOR2, and MetaPredict revealed extensive intrinsically disordered regions (IDRs) (Figure 2B). This prediction was further consolidated by AlphaFold structural modeling (MobiDB consensus), which indicated that 98.8% of C9ORF50 lacks stable tertiary folds, a characteristic that precludes conventional domain mapping. Biophysical characterization confirmed hallmark IDP signatures: both human and mouse C9ORF50 exhibited low order‐promoting residue (OPR) content (human: 27.1%; mouse: 28.6%) and high disorder‐promoting residue (DPR) enrichment (human: 62.2%; mouse: 57.5%), values comparable to canonical IDPs like alpha‐synuclein, osteopontin, emerin, and TFIP11 (Figure 2C). Charge‐hydropathy analysis further classified C9ORF50 within the disordered protein regime (human: net charge = 0.0680, hydropathy = 0.4228; mouse: net charge = 0.0800, hydropathy = 0.3987) (Figure 2D). These features suggest C9ORF50 is a canonical IDP.

Given IDRs' role in LLPS, we reconstituted C9ORF50 phase separation behavior in vitro. Purified mouse and human C9ORF50‐GFP (5–40 μM) formed spherical condensates that fused into larger structures over time and were concentration‐dependent (Figure 2E). FRAP assays in living cells revealed dynamic nuclear puncta of C9ORF50‐GFP exhibiting fusion events, with 76.9% (human) and 82.4% (mouse) fluorescence recovery within 60 s post‐bleaching (Figure 2F). Super‐resolution microscopy showed that C9ORF50 was colocalized with coilin‐positive Cajal bodies (CBs) and SMN‐positive Gemini of Cajal bodies (GEMs), subnuclear hubs governing spliceosome assembly. Notably, knockout of *C9orf50* led to a reduction in the formation of coilin‐positive Cajal bodies and SMN‐positive Gemini of Cajal bodies, while their structural integrity and subnuclear density were restored upon rescue of *C9orf50* expression (Figure 2G–I). These results suggest that C9ORF50 drives LLPS to form dynamic nuclear condensates which may correlate with spliceosome assembly.

### C9ORF50 interacts with spliceosome components and influences their expression

**Figure 3:**
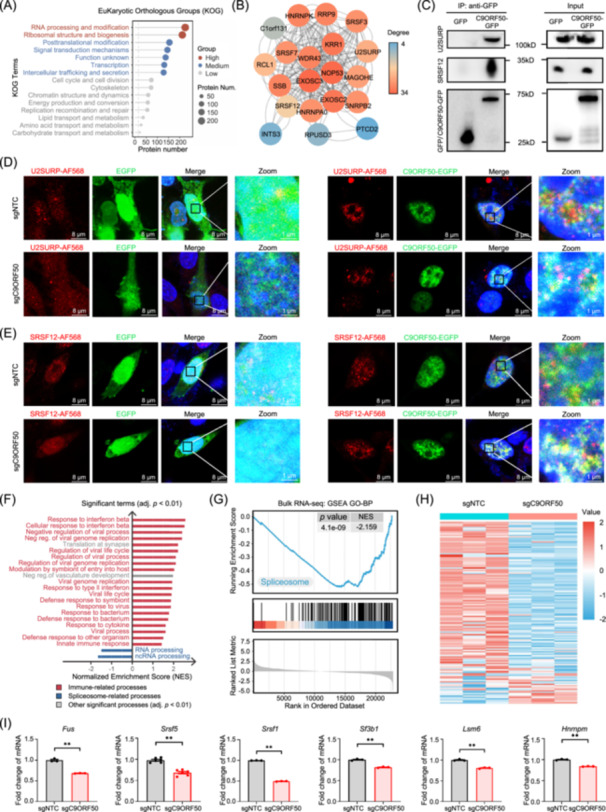
C9ORF50 associates with the spliceosome machinery and regulates spliceosome‐related gene expression. (A) Clusters of Orthologous Groups (COG)/Eukaryotic Orthologous Groups (KOG) enrichment analyses the protein set enrichment of interacting with the C9ORF50. (B) The interaction network that interacted with the C9ORF50. The complex network was constructed by the STRING database, and illustrated with the Cytoscape software. (C) Western blot analysis confirms that both U2SURP and SRSF12 interacted with the C9ORF50. HEK293T cells were transfected with pCMV‐C9ORF50‐GFP or pCMV‐GFP plasmids. Cell lysates were immunoprecipitated with anti‐GFP antibody, followed by immunoblotting analysis with antibodies against U2SURP and SRSF12. SDHA served as loading control. Images representative of 3 biological repeats. Representative immunofluorescence of U2SURP (D) and SRSF12 (E) in MC38 cells under four conditions: upper left, co‐localization of GFP and U2SURP/SRSF12 in MC38‐sgNTC cells; upper right, co‐localization of C9ORF50‐GFP and U2SURP/SRSF12 in MC38‐sgNTC cells; lower left, co‐localization of GFP and U2SURP/SRSF12 in MC38‐sgC9ORF50 cells; lower right, co‐localization of C9ORF50‐GFP and U2SURP/SRSF12 in MC38‐sgC9ORF50 cells. Column 4 displays 5× zoom of boxed regions of Merge. Scale bar, 8 μm. Images representative of 3 biological repeats. (F) Hierarchical clustering of genes significantly changed in MC38‐sgC9ORF50 cells compared with that in MC38‐sgNTC cells by RNA‐seq. (G) Gene set enrichment analysis (GSEA) analyzed the spliceosome pathway genes set in MC38‐sgC9ORF50 cells compared with that in MC38‐sgNTC cells. (H) Heatmap of the alternative splicing regulation genes in MC38‐sgC9ORF50 cells compared with that in MC38‐sgNTC cells. Gene names are provided in Table S4. (I) Expression of *Fus*, *Srsf5*, *Srsf1*, *Sf3b1*, *Lsm6*, and *Hnrnpm* in MC38‐sgC9ORF50 cells versus MC38‐sgNTC cells was measured by qRT‐PCR. **p < 0.01 by Student's *t* test. *n* = 3, Data are shown as mean ± SEM.

To comprehensively characterize the intracellular interaction network of C9ORF50, we performed co‐immunoprecipitation followed by mass spectrometry analysis (CoIP‐MS). Following stringent filtering to remove nonspecific interactions, we identified 47 proteins that specifically interacted with C9ORF50 (Figure 3A). Notably, our proteomic profiling revealed significant enrichment of RNA processing and modification factors associated with C9ORF50 (Figure 3A,B, Figure S5A). Subsequent validation through CoIP‐Western blotting analysis confirmed robust interactions between C9ORF50 and multiple spliceosome components, including U2SURP and SRSF12 (Figure 3B,C, Figure S5B). Furthermore, immunofluorescence analysis demonstrated substantial co‐localization of endogenous spliceosome factors, such as U2SURP, SRSF12, SF3B1, and U2AF^65^, with C9ORF50‐GFP puncta within nuclear compartments (Figure 3D,E, Figure S5C,D). Knockout of *C9orf50* disrupted the condensed nuclear foci of U2SURP, SRSF12, SF3B1, and U2AF^65^, causing their dispersion throughout the nucleoplasm. The subnuclear density and structural integrity of these factors were restored upon *C9orf50* re‐expression. These findings collectively suggest that C9ORF50 may orchestrate RNA splicing events through the formation of LLPS‐mediated nuclear condensates.

To systematically assess the functional consequences of *C9orf50* deficiency on RNA splicing, we performed transcriptomic profiling comparing *C9orf50* knockout and NTC MC38 cells. Our analysis identified 841 differentially expressed genes (DEGs), consisting of 510 downregulated and 331 upregulated transcripts in *C9orf50*‐knockout cells (Figure S6A,B). Gene ontology (GO) and Kyoto encyclopedia of genes and genomes (KEGG) analysis revealed a striking dichotomy in functional enrichment: upregulated genes were predominantly associated with immune‐related processes, while downregulated genes showed significant enrichment in mRNA splicing pathways (Figure 3F, Figure S6C–F). Further gene set enrichment analysis (GSEA) confirmed the significant downregulation of genes involved in spliceosome‐related pathways (Figure 3G,H, Figure S7A,B and Table S4). To validate these findings, we performed quantitative PCR (qPCR) analysis of key splicing factors, including *Fus*, *Srsf5*, *Srsf1*, *Sf3b1*, *Lsm6*, and *Hnrnpm*, which consistently demonstrated reduced expression levels in *C9orf50* knockout cells (Figure 3I). RNA immunoprecipitation (RIP)‐qPCR analysis showed that anti‐GFP precipitates from MC38 cells overexpressing C9ORF50‐GFP (versus GFP controls) contained abundant intron‐retained transcripts of *Fus* and *Srsf5*, confirming direct binding to their pre‐mRNAs (Figure S7C). Taken together, these results suggest that C9ORF50 plays a role in spliceosome function through interacting with spliceosome components and influencing the expression of spliceosome genes.

### *C9orf50* knockout is associated with altered spliceosome function and increased aberrant alternative splicing events

**Figure 4:**
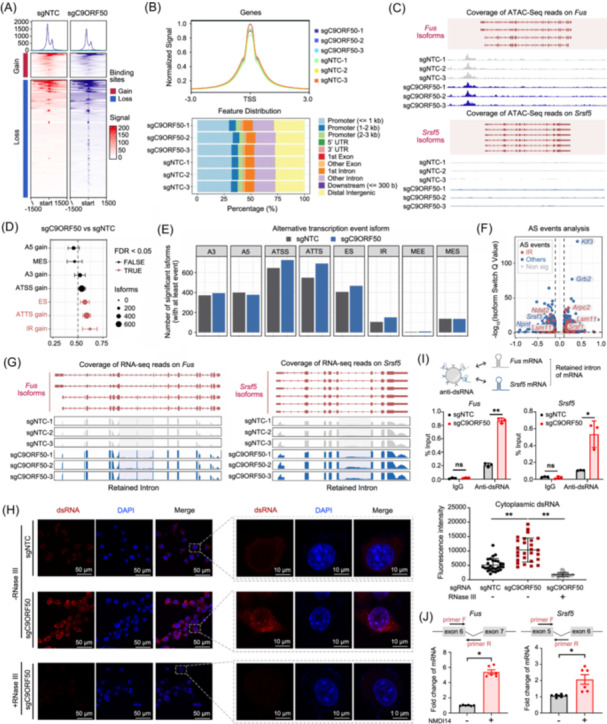
*C9orf50* deficiency impairs spliceosome function via aberrant alternative splicing events in spliceosome‐related genes. (A) Heatmap showing the ATAC‐seq peaks classified on the basis of their changes in MC38‐sgNTC and MC38‐sgC9ORF50 cells. Each row represents a locus (ATAC‐seq peak center ± 1.5 kb), and the red gradient color indicates the ATAC‐seq signal intensity. The numbers above the column indicate the replicates. (B) Metaplot showing the ATAC‐seq signals at TSSs in MC38‐sgNTC and MC38‐sgC9ORF50 cells (above). Annotation of the location of ATAC peaks in MC38‐sgNTC or MC38‐sgC9ORF50 samples to the reference genes (bottom). (C) ATAC‐Seq reads for representative spliceosome components influenced by *C9orf50* knockout. *Fus* (above) and *Srsf5* (bottom) gene were shown. (D) Summary plot showing the enrichment of specific isoform features (consequence) resulting from the observed isoform switching events. The *x*‐axis of the plot shows the fraction of switches having the indicated consequence, where <0.5 means depleted while >0.5 means enriched upon *C9orf50* knockout. (E) Number of detected alternative splicing events among the alternative splicing types. Intron retention (IR), exon skipping (ES), multiple exons skipping (MES), alternative transcription start sites (ATSS), alternative transcription termination sites (ATTS), alternative 5' splice sites (A5), and alternative 3' splice sites (A3). (F) Volcano plot showing the differential isoform fraction (dIF) and the significance of the switching isoforms. (G) Density of RNA‐Seq reads (blue) mapping to exons (red) and introns in the *Fus* and *Srsf5* gene, showing retention of intron (marked by gray shadow). (H) *C9orf50* deficiency induces cytoplasmic dsRNA accumulation. MC38‐sgNTC and MC38‐sgC9ORF50 cells were fixed and visualized by fluorescence microscopy using antibodies against *dsRNA* (red). RNase III treatment used as negative control for dsRNA signal. Scale bars, 50 μm (left), 5 μm (right). Images representative of three experiments. Quantification of cytoplasmic dsRNA signal intensity by ImageJ software. *p < 0.05, **p < 0.01 by Student's *t* test. Data are shown as mean ± SEM, *n* = 34. (I) Schematic diagram of the experimental design (above). RIP‐qPCR analysis of intronic dsRNA enrichment (bottom). dsRNA was immunoprecipitated from MC38‐sgNTC and MC38‐sgC9ORF50 cells using the J2 anti‐dsRNA antibody. Enriched dsRNA was quantified by qPCR using primers targeting intronic regions of *Fus* and *Srsf5*. No significant difference (ns.) (*p* > 0.05), *p < 0.05, **p < 0.01 by Student's *t* test, Data are shown as mean ± SEM, *n* = 3. (J) The qPCR analysis of the mRNA level of genes in MC38‐sgC9ORF50 cells after treated with/without NMDI14. The locations of the designed primers for *Fus* and S*rsf5* genes are shown: forward primer F; backward primer R. Boxes represent exon sequences, black lines represent intronic sequences. **p < 0.01 by Student's *t* test, Data are shown as mean ± SEM, *n* = 3.

To investigate the mechanistic basis of spliceosome gene suppression upon *C9orf50* depletion, we first performed genome‐wide chromatin accessibility profiling using assay for transposase‐accessible chromatin sequencing (ATAC‐seq). Comparative analysis revealed 606 sites with increased accessibility and 1204 sites with decreased accessibility in *C9orf50* knockout cells relative to NTC controls (Figure 4A). However, correlation analysis of chromatin accessibility at promoter–transcription start site (TSS) regions revealed no significant differences between *C9orf50* knockout and NTC cells, suggesting that *C9orf50* ablation does not globally remodel chromatin architecture. (Figure 4B). Notably, multiple spliceosome component genes (*Fus, Srsf5, Srsf1, Sf3b1, Lsm6*, and *Hnrnpm*) whose mRNA levels were significantly downregulated in *C9orf50* knockout cells (Figure 3I), displayed chromatin accessibility patterns indistinguishable from controls (Figure 4C, Figure S8A–D). This discordance between transcriptional output and chromatin state implies the involvement of posttranscriptional regulatory mechanisms in mediating spliceosome gene suppression following *C9orf50* deficiency.

Subsequently, we investigated whether *C9orf50* depletion affects the processing of spliceosome‐related transcripts. Using a differential transcript usage analysis of RNA‐seq data, we identified widespread aberrant alternative splicing events, including Intron retention (IR), exon skipping (ES), and alternative transcription termination sites (ATTS), in *C9orf50* knockout MC38 cells (Figure 4D–F). In spliceosome‐related genes like *Fus* and *Srsf5*, we observed an increased level of reads mapped to intronic regions and exon‐intron boundaries, indicating defective splicing following *C9orf50* knockout (Figure 4G). While we observed intron retention in several spliceosome components, genome‐wide analysis reveals intron retention events also occur in other gene classes, suggesting a broader impact on splicing fidelity (Figure 4D‐G). Given that intron‐retained transcripts are known to form stable dsRNA structures, we hypothesized that the widespread IR observed in *C9orf50*‐knockout cells could be a primary source of immunogenic dsRNA [32]. To test this, we performed immunofluorescence assays to detect cytoplasmic dsRNA. The results revealed a significant accumulation of cytoplasmic dsRNA in *C9orf50* knockout cells, which was abrogated by RNase III treatment, confirming the presence of dsRNA (Figure 4H). Notably, IF staining analyses for dsRNA on mouse tumor tissues robustly demonstrated that *C9orf50* deficiency induces profound dsRNA accumulation (Figure S8E,F), further corroborating the cell‐based findings in vivo. We next determined whether IR events in spliceosome genes directly contribute to dsRNA pools. dsRNA immunoprecipitation (RIP) with J2 antibody followed by qPCR revealed significant enrichment of intronic sequences from *Fus* and *Srsf5* in *C9orf50* knockout cells (Figure 4I). These findings demonstrate that *C9orf50* depletion induces IR in spliceosomal factor transcripts, leading to the formation of stable dsRNA structures derived from unprocessed intronic sequences.

Additionally, IR can induce premature stop codons, triggering nonsense‐mediated mRNA decay (NMD) [33]. Indeed, treatment with the NMD inhibitor NMDI14 further elevated levels of intron‐retaining spliceosome transcripts, suggesting these genes undergo NMD (Figure 4J). Moreover, RNA‐seq results also showed that *C9orf50* knockout MC38 cells upregulated the expression of NMD‐related genes (Figure S7A). These findings demonstrate that *C9orf50* knockout impairs the spliceosome's ability to efficiently process pre‐mRNA, resulting in reduced splicing efficiency and increased intron retention in the RNA profiles.

### *C9orf50* depletion is associated with innate immune activation and cytoplasmic dsRNA accumulation

**Figure 5:**
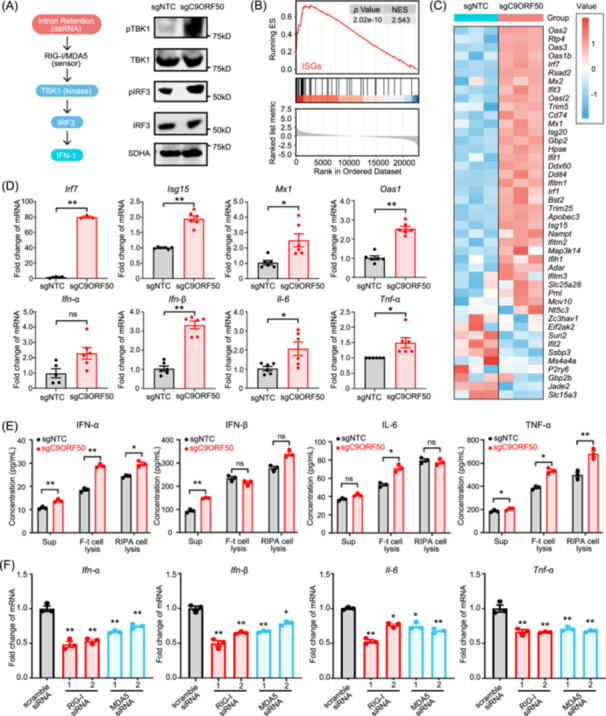
*C9orf50* knockout activates antitumor immunity through cytoplasmic accumulation of double‐stranded RNA. (A) *C9orf50* deficiency leads to increase of the phosphorylation of IRF3 (pIRF3) and TBK1 (pTBK1) in MC38‐sgC9ORF50 cells compared to MC38‐sgNTC cells. Protein levels of pTBK1/TBK1, pIRF3/IRF3, and SDHA were measured by Western blotting analysis. SDHA served as loading control. (B) The enriched gene sets of ISG in MC38‐sgC9ORF50 cells compared with that in MC38‐sgNTC cells are analyzed using GSEA. (C) Heatmap of the ISG in MC38‐sgC9ORF50 cells and MC38‐sgNTC cells. (D, E) *C9orf50* deficiency leads to production of ISG. Gene expression was assayed by qPCR (D). Enzyme‐linked immunosorbent assay (ELISA) validation experiments confirmed the expression in MC38‐sgNTC cells. No significant difference (ns.) (*p* > 0.05), *p < 0.05, **p < 0.01 by Student's *t* test. Data are shown as mean ± SEM, *n* = 3 (E). Cell culture medium, cell lysis (RIPA‐treated or freezing and thawing (F‐t)) was collected, and the secretion levels of cytokines were detected by ELISA. No significant difference (ns.) (*p* > 0.05), *p < 0.05, **p < 0.01 by Student's *t* test. Data are shown as mean ± SEM, *n* = 3. (F) The activation of the antiviral immune signaling pathway upon *C9orf50* knockout is mediated by *RIG‐I* and *MDA5* in MC38 cells. MC38‐sgC9ORF50 cells were transfected with control siRNA (scramble siRNA) or siRNAs targeting *RIG‐I* and *MDA5*, and assessed for *Ifn‐α, Ifn‐β, Il‐6* and *Tnf‐α*. *p < 0.05, **p < 0.01 by Student's *t* test. Data are shown as mean ± SEM, *n* = 3.

Prior studies have shown that cytoplasmic dsRNA is likely detected by dsRNA recognition receptors, such as RIG‐I and MDA5, initiating a signaling cascade that activates TANK binding kinase 1 (TBK1) and IκB kinase ε (IKKε), leading to the phosphorylation of interferon regulatory factor 3 (IRF3) for type I interferon (IFN) expression [34, 35, 36]. Accordingly, western blot analysis confirmed increased phosphorylation of IRF3 and TBK1 following *C9orf50* knockout, indicative of their activation (Figure 5A). Moreover, in subcutaneous tumor tissues derived from *C9orf50*‐knockout cells, phosphorylation of IRF3 was also significantly enhanced (Figure S8G). Key interferon‐stimulated genes (ISGs), including *Irf7, Isg15, Mx1* and *Oas1*, as well as cytokines *Ifn‐α, Ifn‐β, Il‐6*, and *Tnf‐α*, were upregulated in *C9orf50* knockout MC38 cells compared to NTC controls (Figure 5B–D). ELISA results demonstrated a significant increase in the secretion of IFN‐α, IFN‐β, and TNF‐α into the cell culture supernatant. This pro‐inflammatory signature was consistently observed inside the cells, with significantly elevated levels of IFN‐α and TNF‐α across all lysate fractions. Additionally, IL‐6 was found to be specifically increased in the freeze‐thaw lysates (Figure 5E). Additionally, to investigate the potential roles of RIG‐I and MDA5 in activating the antiviral pathway following *C9orf50* knockout, we knocked down both pattern recognition receptors (PRRs) in *C9orf50* knockout MC38 cells using two distinct siRNAs. This knockdown partially reduced cytokine expression (Figure 5F), suggesting that dsRNA sensors contribute to immune activation, though additional pathways may also be involved. Notably, we confirmed that this innate immune activation occurs independently of antigen presentation pathways. Flow cytometric analysis revealed no significant alteration in surface MHC‐I expression following *C9orf50* depletion (Figure S8H). Furthermore, assessment of global ubiquitination levels showed no substantial changes (Figure S8I). Together, these findings indicate that *C9orf50* depletion activates the cytoplasmic dsRNA‐sensing pathway, leading to potent innate immune responses in cancer cells, potentially contributing to antitumor immunity.

### *C9orf50* depletion remodels the TME with increased T cell infiltration

**Figure 6:**
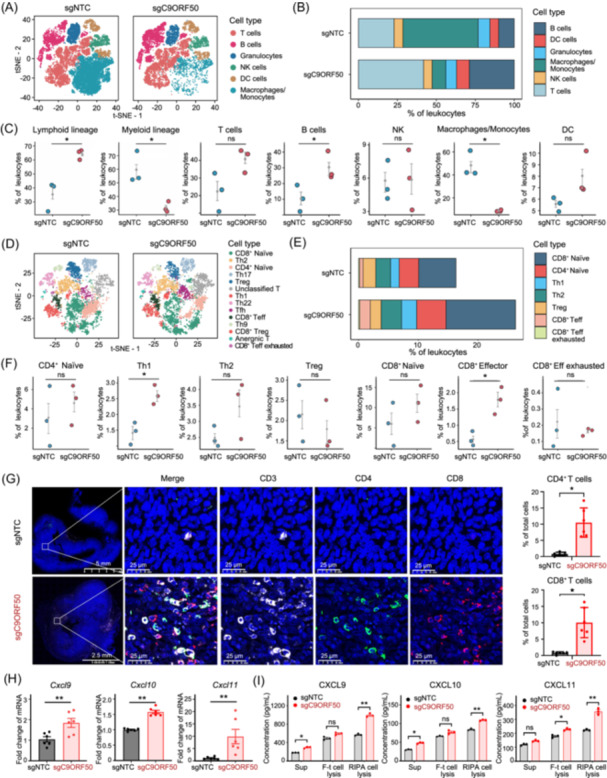
*C9orf50* knockout remodels the tumor microenvironment (TME) into an immune‐stimulatory state. (A) All tumor‐infiltrating immune cells from *C9orf50*‐knockout (sgC9ORF50, *n* = 3 mice) and nontargeting control (sgNTC, *n* = 3 mice) tumor were clustered and showed as t‐distributed Stochastic Neighbor Embedding (tSNE) plots. Six major leukocyte cell types including Macrophages/Monocytes, T cells, B cells, Granulocytes, NK cells, and Dendritic cells are shown. The dendritic cell (DC) population grouped into two major subsets, revealing an architecture comprising conventional DCs (cDCs) and monocyte‐derived DCs (moDCs) [37, 38]. (B) Comparative analysis of immune cell infiltration between *C9orf50*‐deficient and control tumors. Bar plots quantify the relative proportions of tumor‐infiltrating immune subsets, including macrophages/monocytes, T cells, B cells, granulocytes, NK cells, and dendritic cells. (C) Comparing the differentiation potential of cell subpopulations in the sgNTC or sgC9ORF50 groups. The dots and box lines show subgroup scores. *p* values were obtained using a two‐sided Wilcoxon rank‐sum (Mann–Whitney) test with Benjamini–Hochberg correction across subpopulations; *p < 0.05, No significant difference (ns.) (*p* > 0.05). (D) The tSNE plot shows the re‐clustering and re‐annotating T cells and other different cell subsets in the sgNTC or sgC9ORF50 groups. 13 major T cell types are shown. (E) Comparative analysis of T cell subtypes infiltration between *C9orf50*‐deficient and control tumors. Bar plots quantify the relative proportions of tumor‐infiltrating T cell subtypes subsets, including CD8^+^ Naïve, CD4^+^ Naïve, Th1, Th2, Treg, CD8^+^ effector T (CD8^+^ Teff) and exhausted CD8^+^ effector T (CD8^+^ Teff exhausted). (F) Comparing the differentiation potential of cell subpopulations in the sgNTC or sgC9ORF50 groups. The dots and box lines show subgroup scores. *p* values were obtained using a two‐sided Wilcoxon rank‐sum (Mann–Whitney) test with Benjamini–Hochberg correction across subpopulations; *p < 0.05, No significant difference (ns.) (*p* > 0.05). (G) Representative CD3, CD4, and CD8 multicolor immunofluorescence staining in the sgNTC or sgC9ORF50 tumors. Quantification of CD4 and CD8 immunoreactivities. Scale bar, 25 μm. Images representative of three experiments. *p < 0.05 by Student's *t*‐test. Data are shown as mean ± SEM, *n* = 6. (H) *C9orf50* deficiency leads to production of chemokines. Gene expression was assayed by qPCR. **p < 0.01 by Student's *t* test. Data are shown as mean ± SEM, *n* = 6. (I) ELISA validation experiments confirmed the expression in MC38‐sgNTC cells. Cell culture medium, cell lysis (RIPA‐treated or freezing and thawing (F‐t)) was collected, and the secretion levels of chemokines were detected by ELISA. No significant difference (ns.) (*p* > 0.05), *p < 0.05, **p < 0.01 by Student's *t* test. Data are shown as mean ± SEM, n = 3.

To elucidate the impact of *C9orf50* knockout on the immune cell compositions of the TME, we performed an immune landscape profiling of syngeneic MC38 tumors with *C9orf50* knockout versus NTC controls. Flow cytometry analysis revealed a marked increase of CD45^+^ leukocyte infiltration in *C9orf50* knockout tumors (Figure S9A). Subsequent single‐cell RNA sequencing (scRNA‐seq) of the CD45^+^ tumor‐infiltrating leukocytes, yielded a comprehensive transcriptional landscape of 39,544 leukocytes. Unsupervised clustering identified 18 distinct leukocyte clusters. Through deep learning‐based and manual marker‐based annotation, six major leukocyte subsets were identified: macrophages/monocytes, T cells, B cells, granulocytes, NK cells, and dendritic cells (Figure 6A, Figure S9B,C). Analyses of these subsets showed a significant increase in lymphoid infiltration in *C9orf50* knockout tumors (*p* = 0.033), B cells (*p* = 0.048), along with a reduction in myeloid infiltration (*p* = 0.047), especially macrophages/monocytes (*p* = 0.025) (Figure 6B,C). By re‐clustering of T cells, we characterized 11,775 cells across 13 distinct T cell subtypes (Figure 6D, Figure S9D). *C9orf50* knockout led to significant increases in Th1 cells (*p* = 0.021) and CD8^+^ effector T cells (*p* = 0.025) compared to controls (Figure 6E,F), without significant changes in Treg cells.

Further differential expression analysis (DEA) in CD8^+^ T cells revealed an enrichment of genes involved in T cell receptor signaling pathway and immune response‐regulation (Figure S9E). Similarly, DEGs in Th1 and Th2 cells were implicated in biogenesis of ribonucleoprotein complex and regulation of mRNA processing (Figure S9E). Moreover, macrophage infiltration, particularly the M2 subtype, was significantly reduced in *C9orf50* knockout tumors (Figure S9F–I). These results suggest that *C9orf50* knockout may reprogram the TME from an immune‐refractory state to an immune‐stimulatory state, characterized by enhanced infiltration of effector T cells and reduced immunosuppressive myeloid cells, especially M2 macrophages. This immune reprogramming fosters an immune‐stimulatory TME, thereby enhancing susceptibility to immune‐mediated clearance. To validate these findings from scRNA‐seq, we performed immunofluorescence staining for CD8 and CD4 in mouse syngeneic tumors, which showed significantly higher infiltration of CD8^+^ and CD4^+^ T cells in *C9orf50* knockout tumors compared to NTC control (Figure 6G). In addition to the pronounced increase in T cell infiltration, deep immunoprofiling of the TME revealed concomitant shifts in myeloid and B cell compartments. While the relative proportion of macrophages decreased, likely because of the marked expansion of T and B lymphocytes, GSEA revealed that these remaining macrophages underwent a functional shift. This shift away from an immunosuppressive phenotype was characterized by downregulation of specific genes (Figure S10A–C). Similarly, B cells exhibited not only an increase in proportion but also transcriptional signatures suggestive of enhanced activation and humoral immunity (Figure S10D–F). These changes, consistent with an overall immunostimulatory reprogramming of the TME, likely provide ancillary support to the dominant antitumor effects mediated by T cells.

Given that type I ISGs include chemokines, such as CXCL9, CXCL10, and CXCL11, are crucial for T cell recruitment, we hypothesized that *C9orf50* knockout could enhance T cell infiltration by altering chemokine production [39]. Indeed, *C9orf50* knockout in MC38 cells led to upregulation of T cell chemo‐attractants, specifically CXCL9, CXCL10, and CXCL11, as confirmed by RNA‐seq, qPCR, and ELISA (Figure 6H,I, Figure S11A–C). Furthermore, knockdown of *RIG‐I* and *MDA5* in *C9orf50*‐knockout cells significantly downregulated the expression of these chemokines, suggesting that this effect is mediated through dsRNA‐sensing PRRs (Figure S11D). These findings indicate that *C9orf50* knockout promotes T cell infiltration by reshaping the TME through elevated expression of T cell chemo‐attractants.

### Elevated *C9ORF50* expression in tumors correlates with poor clinical outcomes

**Figure 7:**
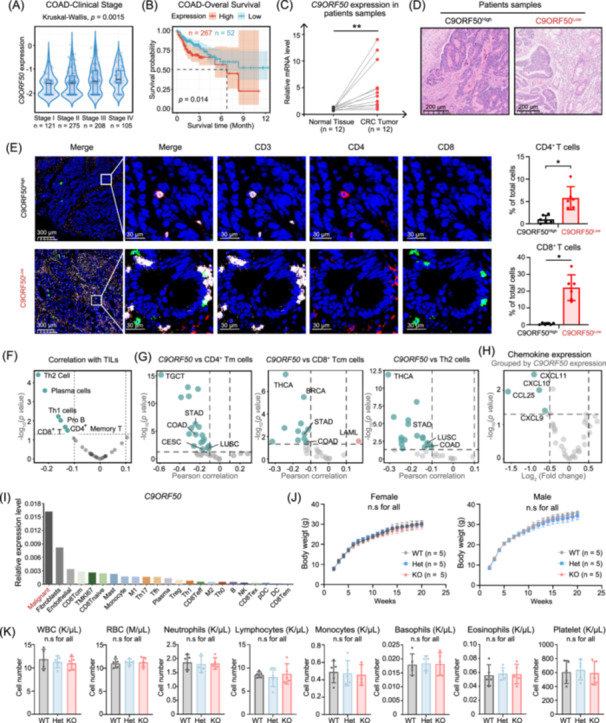
*C9ORF50* correlates with adverse clinical outcomes and exhibits tumor‐associated expression. (A) Expression levels of *C9ORF50* in colorectal cancer patients across different clinical stages. Data were obtained from the TCGA pan‐cancer database (colorectal cancer cohort), including *n* = 121 (Stage I), *n* = 275 (Stage II), *n* = 208 (Stage III), and *n* = 105 (Stage IV) patients. (B) Kaplan–Meier analysis of overall survival in colorectal cancer patients stratified by *C9ORF50* expression levels (log‐rank test). Patients were classified into low (*n* = 52) and high (*n* = 267) expression groups based on optimal cut‐off values. (C) *C9ORF50* gene expression differences between 12 colorectal cancer patients' samples. **p < 0.01 by Student's *t* test. Data are shown as mean ± SEM, *n* = 12. (D) Representative H&E images of tumors in (C). Scale bar, 200 μm. Images representative of 3 biological repeats. (E) Representative CD3, CD4 and CD8 multicolor immunofluorescence staining in *C9ORF50* expressed high (C9ORF50^High^; above) or low (C9ORF50^Low^; bottom) human colorectal cancer tumors samples. Quantification of CD4 and CD8 immunoreactivities. Scale bar, 30 μm. Images representative of 3 experiments. *p < 0.05, **p < 0.01 by Student's *t* test. Data are shown as mean ± SEM, *n* = 3. (F) Volcano plot of the Spearman correlation between *C9ORF50* mRNA expression and tumor infiltration immune cells (TILs) abundance in colorectal cancer. Green dots indicate immune cells significantly negatively correlated with *C9ORF50* mRNA expression (adjusted *p* < 0.05). (G) Volcano plot of the Spearman correlation between *C9ORF50* mRNA expression CD4^+^ T memory cells, CD8^+^ T, and Th2 abundance in multiple cancer types (Stomach adenocarcinoma (STAD), Lung squamous cell carcinoma (LUSC), Cervical squamous cell carcinoma and endocervical adenocarcinoma (CESC), etc.). Red dots indicate immune cells significantly positively correlated with *C9ORF50* mRNA expression (adjusted *p* < 0.05). Green dots indicate immune cells significantly negatively correlated with *C9ORF50* mRNA expression (adjusted *p* < 0.05). (H) Volcano plot of the Spearman correlation between *C9ORF50* mRNA expression and chemokines mRNA expression in colorectal cancer. Green dots indicate chemokines expression is significantly negatively correlated with *C9ORF50* mRNA expression (adjusted *p* < 0.05). (I) The expression profile of *C9ORF50* in malignant cells, immune cells, and healthy tissues was analyzed using the Immune‐related CRISPR screen Analyzer of Functional Targets (ICRAFT) platform. (J) Body weights of mice with indicated *C9orf50* genotypes. No significant (ns.) by one‐way ANOVA with Tukey's post hoc test between any two groups at any time for male or female. Wild‐type mice, WT; *C9orf50* ^−/+^ heterozygote mice, Het; *C9orf50* knockout mice, KO. (K) Peripheral blood profiles of 8‐week mice of indicated genotype. Wild‐type mice, WT; *C9orf50* ^−/+^ heterozygote mice, Het; *C9orf50* knockout mice, KO. No significant difference (n.s.) by one‐way ANOVA with Tukey's post hoc test. Data are shown as mean ± SEM, *n* = 5.

To explore the clinical relevance of our findings, we analyzed *C9ORF50* expression in human cancer cohorts (Figure S12A). Elevated *C9ORF50* expression was significantly associated with advanced clinical staging in colon adenocarcinoma (Figure 7A). Subsequent survival analysis showed that C9ORF50^high^ cohort had significantly worse overall survival compared to C9ORF50^low^ cohort (Figure 7B, Figure S12B). Furthermore, qPCR analysis of 12 matched pairs of colorectal cancer tissues and adjacent noncancerous tissues revealed a significant upregulation of *C9ORF50* in malignant tissues (Figure 7C). To elucidate the relationship of *C9ORF50* and immune infiltration in the TME, we performed histology and immunofluorescence analysis on human colorectal cancer samples. Histological analysis showed that C9ORF50^low^ tumors exhibited a lower cell density and pronounced leukocyte infiltration compared to C9ORF50^high^ tumors (Figure 7D). C9ORF50^low^ tumors also showed elevated levels of dsRNA and increased phosphorylation of IRF3, suggesting enhanced activation of innate immune signaling pathways (Figure S12C,D). Further multiplexed immunofluorescence analysis confirmed that C9ORF50^low^ tumors had significantly higher levels of infiltrated CD8^+^ and CD4^+^ T cells compared to the C9ORF50^high^ counterparts (Figure 7E). This observation was further corroborated by TCGA data set analysis, which revealed a consistent inverse correlation between *C9ORF50* expression and the infiltration of various lymphoid cell populations, including CD8^+^ T cells, Th1, Th2, CD4^+^ memory T cells, and plasma cells across multiple human cancer types (e.g., STAD, LUSC, CESC; Figure 7F,G). In addition, TCGA analysis demonstrated an inverse correlation between *C9ORF50* expression and the transcriptional levels of several key T cell chemokines (CXCL9, CXCL10, and CXCL11), consistent with our mechanistic findings (Figure 7H). Collectively, these findings establish a compelling association between *C9ORF50* overexpression and the establishment of an immunosuppressive TME, ultimately contributing to poor patient survival outcomes across multiple cancer types.

Differential expression profiling via the Immune‐related CRISPR screen Analyzer of Functional Targets (ICRAFT) platform highlighted a tissue‐restricted expression signature of *C9ORF50*, with predominant abundance in malignant cells and minimal expression in immune cells and healthy tissues, underscoring its potential as a pharmacologically targetable molecule with limited off‐target effects (Figure 7I) [40]. Accordingly, *C9orf50* knockout mice were born at the expected Mendelian ratio and displayed no overt developmental or physiological abnormalities. Detailed examination of body weight trajectories and blood cell profiles showed no significant differences between knockout and wild‐type mice (Figure 7J,K), indicating that *C9orf50* is dispensable for normal animal development and physiological homeostasis. Collectively, the relatively tumor‐specific expression pattern, combined with the non‐essential nature of *C9ORF50* in normal physiology, strongly suggests that targeted inhibition of this protein may represent a promising therapeutic strategy with inherently constrained potential deleterious effects. These results provide a compelling rationale for further investigation of *C9ORF50* as a potential therapeutic target in cancer.

### *C9orf50*‐targeting RNA interference suppresses cancer growth

**Figure 8:**
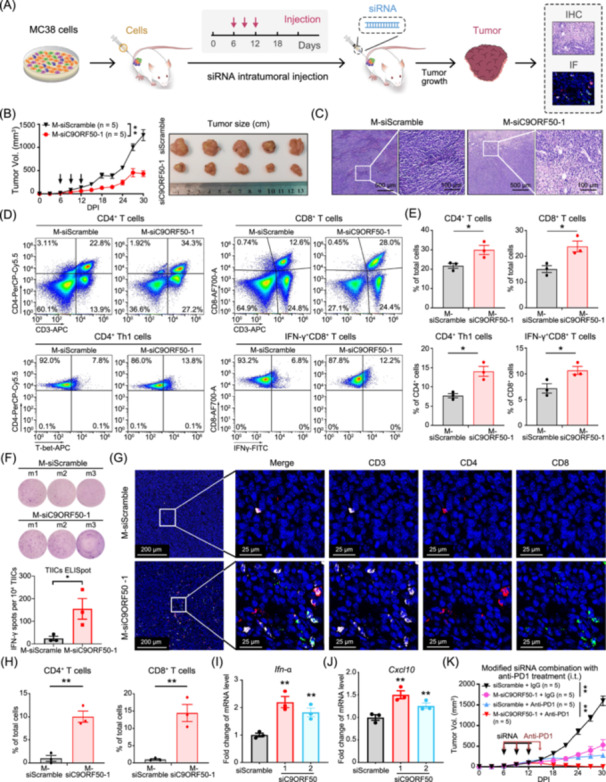
Therapeutic targeting of *C9orf50* via RNA interference suppresses cancer progression. (A) Schematic illustration of the immunotherapy mediated by siRNA in vivo. (B) Tumor growth curves after intratumoral injection (i.t.) of cholesterol‐C9ORF50‐siRNA (M‐siC9ORF50‐1, 5 mice/group) or cholesterol‐scramble‐siRNA (M‐siScramble, 5 mice/group). **p < 0.01 by two‐way ANOVA with Holm‐Sidak's multiple comparisons test. Photographs of tumors derived from each treatment group. (C) H&E‐stained tumor slices after different treatments. Scale bar, 500 or 100 μm. Images representative of 3 biological repeats. (D) Representative infiltration of CD4^+^ T cells, CD8^+^ T cells, CD4^+^ Th1 cells and IFN‐γ^+^CD8^+^ T cells in the TME of cholesterol‐C9ORF50‐siRNA‐1 (M‐siC9ORF50) or M‐siScramble tumors analyzed by flow cytometry. (E) Quantification of CD4^+^ T, CD8^+^ T, CD4^+^ Th1 and IFN‐γ^+^CD8^+^ T cells of (D). *p < 0.05 by Student's *t‐*test. Data are shown as mean ± SEM, *n* = 3. (F) Representative images (above) and quantification (bottom) of IFN‐γ ELISpots performed on TIICs isolated from M‐siC9ORF50‐1 or M‐siScramble tumors. *p < 0.05 by Student's *t*‐test. Data are shown as mean ± SEM, *n* = 3. (G) Representative CD3, CD4, and CD8 multicolor immunofluorescence staining in cholesterol‐C9ORF50‐siRNA (M‐siC9ORF50‐1) or M‐siScramble tumors. Scale bar, 200 and 25 μm. Images representative of three biological repeats. (H) Quantification of CD3, CD4, and CD8 immunoreactivities. **p < 0.01 by Student's *t* test. Data are shown as mean ± SEM, *n* = 3. (I, J) Knockdown of *C9orf50* with siC9ORF50‐1 or siC9ORF50‐2 significantly upregulated mRNA expression of *Tnf‐α* (I) and chemokines *Cxcl10* (J) in MC38 cells. The data represent the mean ± SEM, *n* = 3. **p < 0.01 by Student's *t* test. (K) Tumor growth curves intratumoral (i.t.) injection of M‐siC9ORF50‐1 or siScramble control (5 mice/group), combined with intraperitoneal (i.p.) administration of 200 μg anti‐PD1 antibody or rat IgG2a isotype control every 3 days (four total doses). **p < 0.01 by two‐way ANOVA with Holm‐Sidak's multiple comparisons test.

To evaluate the potential of *C9orf50* as a cancer therapeutic target, we investigated the antitumor effects of *C9orf50* inhibition via direct intratumoral administration of *C9orf50* siRNA (siC9ORF50) in MC38 tumor‐bearing mice (Figure 8A). After identifying effective siRNAs targeting *C9orf50* (Figure S13A), we conducted intra‐tumoral administration of cholesterol‐modified, in vivo stable *C9orf50* siRNAs (M‐siC9ORF50‐1/‐2) at a dose of 1 mg/kg. Tumor‐bearing mice treated with *C9orf50* siRNA showed a significant reduction in tumor burden compared to those treated with scramble siRNA control (Figure 8B, Figure S13B). Histopathological analysis showed reduced tumor cell density in tumors treated with *C9orf50* siRNA (Figure 8C, Figure S13C). Flow cytometry analysis demonstrated that *C9orf50* interference significantly increased tumor infiltration of both CD4^+^ and CD8^+^ T cells (Figure 8D,E), with significant expansion of the Th1 and IFN‐γ‐producing CD8^+^ T cell subset. ELISpot assays showed an increase in IFN‐γ‐producing TIICs following treatment with M‐siC9ORF50‐1, compared to the scramble siRNA control (Figure 8F). Although an upward trend in IFN‐γ secretion by tumor‐infiltrating immune cells (TIICs) was observed after M‐siC9ORF50‐2 treatment, the difference did not reach statistical significance (*p* = 0.17) (Figure S13D). These findings were further supported by immunofluorescence staining, which revealed a significantly increased spatial density of both CD4^+^ and CD8^+^ T cells within the TME following *C9orf50* interference (Figure 8G, H). In parallel, *C9orf50* silencing induced robust upregulation of immunostimulatory cytokines (*Ifn‐α/β, Il‐6, Tnf‐α*) and T cell‐recruiting chemokines (*Cxcl9, Cxcl10, Cxcl11*) in the TME (Figure 8I,J, Figure S13E). Although unmodified *C9orf50* siRNA also exhibited a detectable antitumor effect, it was less potent compared to cholesterol‐modified siRNA (Figure S13F–I). Importantly, combination therapy involving cholesterol‐modified siC9ORF50 and anti‐PD1 antibody resulted in synergistic suppression of tumor growth, exceeding the efficacy of either treatment alone (Figure 8K). Collectively, these results suggested that *C9orf50* represents a promising target for cancer therapy.

## DISCUSSION

In this study, we conducted a genome‐wide CRISPR knockout screen using a high immunogenic MC38‐Cas9 colorectal cancer model in immunocompetent C57BL/6 mice to identify cancer cell‐intrinsic regulator of immune evasion. This screen led to the identification of well‐established oncogenes such as *Alk* and *Spi1*, as well as several previously unrecognized genes [30, 31]. Among them, we identified *C9ORF50*, a previously uncharacterized gene whose knockout resulted in significant tumor regression and improved survival. Examination of clinical samples revealed that *C9ORF50* expression is inversely correlated with cancer patient prognosis. While *C9ORF50* has not been functionally characterized, prior studies have linked its aberrant DNA methylation to early diagnostic biomarkers for colorectal and gastric cancers, suggesting its involvement in gastrointestinal cancers [41, 42]. While these methylation studies highlight the clinical relevance of *C9ORF50* dysregulation in cancer detection, our functional data establish its direct pro‐oncogenic role in tumor progression, suggesting that its epigenetic silencing in accessible biospecimens may reflect field cancerization or secondary events rather than tumor‐suppressive function.

Our research reveals that C9ORF50, a canonical IDP devoid of stable tertiary structures, exhibits LLPS properties and is involved in RNA splicing regulation. This process enables the dynamic assembly of spliceosome components into membraneless condensates, thereby regulating splicing efficiency [43, 44, 45]. Similar mechanisms have been observed in other IDPs, such as EWSR1‐FLI1, which exploits phase separation to hijack transcriptional programs, resulting in oncogenic chimeric transcripts that promote malignant transformation [46, 47]. While C9ORF50 exhibits LLPS properties in vitro and forms dynamic nuclear condensates that colocalize with spliceosome components, we have not formally demonstrated that LLPS is functionally required for its splicing regulatory role. Future studies using LLPS‐defective mutants that retain protein‐protein interactions will be necessary to establish this causal link. Beyond the biophysical mechanism, we also sought to determine whether IDP‐mediated splicing directly influences antitumor immunity. Here, we demonstrate that *C9ORF50* deficiency disrupts RNA splicing fidelity, leading to accumulation of intron‐containing transcripts and cytoplasmic aberrant dsRNA, which in turn triggers antiviral dsRNA‐sensing pathways and extrinsic apoptosis. This disruption remodels the TME, enhancing Th1 and CD8^+^ T cell infiltration, partially through the upregulation of T cell recruiting chemokines. Further analysis of clinical samples revealed a significant inverse correlation between *C9ORF50* expression and adaptive immune cell infiltration and patient prognosis, reinforcing its potential as a cancer therapeutic target. Notably, therapeutic targeting of *C9ORF50* via intratumoral administration of siRNA led to a dramatic reduction in tumor burden, underscoring its potential as a novel immunotherapy target.

While our data support a role for dsRNA‐mediated immune activation via *RIG‐I/MDA5* (Figure 5F), the incomplete reduction of cytokine production upon *RIG‐I/MDA5* knockdown suggests that additional mechanisms may contribute to immune activation. These could include changes in *ADAR1* expression that unmask endogenous dsRNA, activation of endogenous retroviruses independent of splicing defects, direct effects on NF‐κB or interferon regulatory pathways, or mitochondrial stress and dsRNA release. The relative contributions of these pathways warrant further investigation.

Alternative splicing dysregulation is a common characteristic of cancer, affecting tumor progression, metastasis, and therapeutic response [19, 48, 49]. Small molecules targeting the spliceosome have gained prominence as a promising anticancer treatment. Previous research has demonstrated that the inhibition of *U2 snRNP*‐associated spliceosomes components leads to alterations in splicing and gene expression of transcripts that are enriched for RNA‐processing factors. This finding establishes a connection between fundamental splicing mechanisms and their effectors with modulators of alternative splicing and RNA processing [50]. Based on our data, we propose that *C9ORF50* inhibition impairs phase separation within the spliceosome complex, compromising pre‐mRNA splicing, especially the essential spliceosome components like *Fus*, *Srsf5*, *Srsf1*, *Sf3b1*, *Lsm6*, and *Hnrnpm*. This disruption creates a feedforward loop, exacerbating spliceosome dysfunction and inducing widespread splicing alternations that ultimately enhances antitumor immune responses.

A fundamental challenge in precision oncology lies in overcoming the off‐target toxicity of systemically administered therapies, which remains a major barrier to clinical translation for many targeted therapies [51]. This underscores the critical need to develop either tissue‐specific delivery platforms or molecular targets with intrinsically restricted function patterns. *C9ORF50* emerges as an exceptionally promising candidate for cancer therapy in this regard, exhibiting: (i) pronounced tumor specificity with negligible expression in immune cells and healthy tissues; (ii) predominant localization to the gastrointestinal tract, brain, and kidneys while being virtually absent in lymphoid organs (spleen, thymus) and other immune‐rich tissues (liver, lung) [52]; (iii) dispensability for normal development and physiological homeostasis; (iv) strong association with poor clinical outcomes. These characteristics collectively suggest that pharmacological inhibition of *C9ORF50* may offer an unusually favorable therapeutic window with intrinsically limited off‐target consequences. While *C9orf50* knockout mice develop normally and display no overt physiological abnormalities (Figure 7J,K), several safety concerns warrant systematic evaluation. First, previous literature reports testis expression of *C9ORF50*, raising potential concerns about reproductive toxicity that will require formal assessment. Second, clinical experience with other splicing inhibitors (e.g., E7107, H3B‐8800) has revealed organ‐specific toxicities not predicted by preclinical models, highlighting the need for comprehensive safety evaluation including nonhuman primate studies. Third, while tumor‐infiltrating lymphocytes increase after *C9ORF50* inhibition, we have not assessed whether *C9ORF50* loss impairs normal T cell activation or effector functions. Since splicing is required for T cell responses, this requires careful evaluation. Finally, the therapeutic window between tumor suppression and normal tissue toxicity needs rigorous quantification across multiple endpoints before clinical translation can be considered.

While this study provides substantial insights into the role of *C9ORF50* in splicing regulation and antitumor immunity, several important questions remain to be addressed in future research: (i) Deeper structural characterization of its disordered architecture remains essential. As *C9ORF50* is a paradigmatic IDP, conventional approaches were largely ineffective for mapping its functional binding regions through domain‐specific mutagenesis. With advances in de novo IDR‐targeting protein design technology, this methodology will leverage conformation‐preserving perturbation to precisely decode the IDR‐mediated C9ORF50–splicing factors interaction mechanism [53]. (ii) Tumor cells may rely on *C9ORF50* for spliceosome assembly in a way that normal tissues do not, suggesting a unique dependency on distinct splicing mechanisms in malignancies. Moving forward, systematic profiling of tumor‐specific spliceosomal architectures, such as core complex composition, spatiotemporal assembly dynamics of snRNPs and sca/snoRNPs, regulatory factor expression, and pre‐mRNA substrate selectivity, could reveal novel targetable vulnerabilities unique to cancer cells. (iii) Tumor suppression operates predominantly through immune‐mediated mechanisms rather than direct proliferative arrest: intronic dsRNA accumulation triggers extrinsic apoptosis without compromising proliferation, whereas ERV‐derived dsRNA suppresses growth [32, 54, 55]. Further investigations are needed to elucidate the precise mechanisms underlying the differential effects of intron‐derived versus ERV‐derived dsRNA on apoptosis and proliferation. (iv) The MC38 cell line used in this study carries wild‐type KRAS. Future work should explore the function and immunomodulatory effects of *C9ORF50* in KRAS‐mutant colorectal cancer models to determine whether its role remains conserved across different genetic backgrounds.

## LIMITATIONS AND FUTURE DIRECTIONS

This study proposes *C9ORF50* as a splicing regulator that promotes antitumor immunity through liquid‐liquid phase separation, outlining a mechanistic pathway from LLPS disruption to immune activation via splicing defects, intron retention, and dsRNA accumulation. While this model provides a framework for understanding *C9ORF50* function, several aspects require further validation to establish definitive causality. Most critically, rescue experiments using sgRNA‐resistant *C9ORF50* cDNA would determine whether the observed phenotypes specifically result from *C9ORF50* loss rather than clonal variation or off‐target effects. Furthermore, to directly test the functional requirement of phase separation, future studies should employ LLPS‐deficient mutants that disrupt condensate formation while maintaining protein interaction capability. If such mutants fail to rescue the splicing defects and immune phenotypes, this would provide conclusive evidence that LLPS itself, rather than merely protein‐protein interactions, drives *C9ORF50* function.

The basis for the apparent selectivity of intron retention in spliceosome components remains unclear. This pattern could indicate either specific recognition of these transcripts through particular sequence or structural features by *C9ORF50*, or alternatively, heightened intrinsic sensitivity of spliceosome genes to global splicing impairment. Resolving this distinction will require comprehensive genome‐wide analyses, including detailed characterization of affected transcripts and systematic identification of potential *C9ORF50* binding motifs.

Another key mechanistic gap concerns how intron‐retained transcripts evade nuclear quality control to reach the cytoplasm. Normally, such immature RNAs are retained in the nucleus or degraded by surveillance mechanisms including nonsense‐mediated decay. The pathway through which these transcripts escape nuclear detention and undergo export, which enables cytoplasmic dsRNA formation and immune activation, remains undefined. Elucidating this process could uncover novel regulatory mechanisms in RNA quality control with potential therapeutic applications, possibly enabling strategies to enhance immunogenicity through modulated nuclear retention or promoted cytoplasmic accumulation of intron‐retained transcripts.

From a translational perspective, significant challenges persist. Although intratumoral RNAi administration demonstrates proof‐of‐concept efficacy, systemic delivery to solid tumors remains a major hurdle. A critical future direction is to develop advanced delivery systems ranging from lipid nanoparticles, exosomes, and targeted conjugates to alternative approaches such as small‐molecule inhibitors or PROTAC degraders targeting C9ORF50. Additionally, several clinical questions demand resolution regarding optimal patient selection criteria, predictive biomarkers, rational combination strategies with immune checkpoint inhibitors, refined dosing regimens and treatment sequencing, and potential resistance mechanisms. Furthermore, systematic evaluation across diverse cancer models with varied genetic backgrounds, including different *KRAS*, *TP53,* and *MYC* status, along with different mutational burdens and microsatellite instability patterns, will be essential to define the therapeutic scope of *C9ORF50* inhibition and advance this approach toward clinical application.

## CONCLUSION

In summary, our study identifies *C9ORF50* as a previously uncharacterized spliceosome‐associated protein and a candidate target for cancer immunotherapy. Targeting *C9ORF50* remodels the TME by enhancing immune cell infiltration in poorly infiltrated tumors, thereby increasing their susceptibility to immune‐mediated clearance. The relatively tumor‐enriched expression pattern and non‐essential role in normal mouse physiology suggest *C9ORF50* as a promising target for further therapeutic development, though a comprehensive safety evaluation will be necessary. Given the conserved nature of the phase separation domain of *C9ORF50* across vertebrate species, further studies should explore its involvement in other splicing‐related pathologies. Further investigation is warranted to assess the potential compensatory effects of paralogs, such as members of the *C9ORF50* family, as well as the long‐term implications of chronic disturbances in spliceosome function. Moreover, developing humanized models incorporating patient‐derived xenografts will be essential to more accurately validate these findings in clinically relevant settings.

## METHODS

### Animals

Six‐ to eight‐week‐old C57BL/6 and nude mice in both sexes were sourced from commercial vendors and used as transplant hosts. All animals were maintained in standard individually ventilated, pathogen‐free conditions, with a light–dark cycle of 12 h and temperature around 21–25°C, humidity of 40%–60%. Mice were randomly divided into 5 mice per cage. All animals' experiments follow ARRIVE guidelines and followed strict randomization.

### *C9orf50* knockout mice construction

CRISPR/Cas genome editing was used to create *C9orf50* knockout mice, targeting the 5' upstream sequence and intron4. Cas9 mRNA and gRNA, synthesized through in vitro transcription, were injected into fertilized eggs to generate the knockouts. Founders were genotyped using PCR and DNA sequencing, and positive ones were bred for the next generation, confirmed by the same methods. Genomic PCR of tail DNA was used for offspring genotyping, and all mice were compared to wild‐type littermates of the same gender. Peripheral blood profiles were measured using a Hemavet 950 system (DREW Scientific).

### Subcutaneous tumor models

For subcutaneous tumor model, MC38, E0771, Pan02, B16‐F10, LLC, and Hepa cells were injected subcutaneously into each C57BL/6 or Nu/Nu mouse in the right flanks at 2 × 10^6^ cells per flanks, respectively. After inoculation, the tumor‐bearing mice were observed, and tumor size was monitored every 3 days by using vernier callipers with cage labels blinding. The following formula was used to estimate tumor size (all measurements in mm): volume = (4/3) × π × (length/2) × (width/2) × (depth/2). After mice were euthanized, the tumors were removed and weighed.

### Orthotopic mouse models

Male C57BL/6 mice (aged 6–8 weeks) were anesthetized and subjected to a small abdominal incision to expose the intestine. Under microscopic visualization, a 50 µL suspension containing 2 × 10^5^ cells was injected into the intestine wall. The intestine was then returned to the peritoneal cavity, and the incision was closed with sutures. Mice were monitored for recovery and provided with appropriate analgesics. They were observed for any signs of distress or complications. Tumor growth was assessed at the end of the study period, typically 9 days postimplantation, by euthanizing the mice and performing necropsies to examine tumor progression. Tumor volume measurements were performed by independent researchers who were blinded as to treatment group assignment.

### The AOM/DSS‐induced colitis‐associated intestinal carcinogenesis model

The AOM/DSS‐induced colitis‐associated intestinal carcinogenesis model was established as previously described [56]. Briefly, wild‐type of *C9orf50* ^−/−^mice received an intraperitoneal injection of AOM (10 mg/kg body weight) on day 0. This was followed by one cycle of DSS (2% w/v) administered in drinking water for 14 days, starting on Day 7. All mice were euthanized at week 11 after AOM injection, and colon tissues were collected for subsequent examination.

### Cell lines

All cell lines, including HEK293T cells, MC38 (colorectal cancer cells), Hepa1‐6 (Hepatoma cells), Pan02 (pancreatic cancer cells), E0771 (triple‐negative breast cancer cells), and B16F10 (melanoma cells), were obtained from the National Infrastructure of Cell Line Resource (Beijing, China) and authenticated by STR profiling. All cells were cultured in Dulbecco's Modified Eagle Medium or Roswell Park Memorial Institute (RPMI) 1640 supplemented with 10% fetal bovine serum (FBS), 1% Penicillin‐Streptomycin (Penstrep), and maintained in a humidified CO_2_ incubator set to 5% CO_2_ at 37°C. Regular testing using a Mycoplasma Detection Kit (Thermo Fisher Scientific) or conventional PCR ensured that all cell lines were free of Mycoplasma contamination. To mitigate the risk of phenotypic drift, all cell lines were cryopreserved in multiple aliquots upon receipt.

### Genetic CRISPR knockout cells

Target gene knockout cells were generated using lentivirus‐mediated CRISPR‐Cas9 technology. sgRNAs specific for target genes, including *C9orf50*, as well as nontargeting control sgRNAs (sgNTC), were designed using the online CRISPR design tool (F. Zhang lab, MIT, Boston, MA). The sgRNA sequences were cloned into plentiCRISPR‐v2 using the standard protocol. To generate the knockout cell lines, tumor cells were initially transduced with a lentiviral Cas9 to establish Cas9‐stably expressing cell lines. These Cas9 stably expressing cell lines were then transduced with lentiviruses carrying either the sgC9ORF50 or sgNTC expressing sequences, generating *C9orf50*‐knockout cells and NTC cells, respectively.

### Genome‐wide CRISPR‐Cas9 knockout screen *in vivo*

The genome‐wide mBrie CRISPR‐knockout (KO) pooled library was purchased from Addgene and amplified according to the supplier's protocol. Cas9‐expressing MC38 (MC38‐Cas9) cells were transduced with the lentiviruses carrying mBrie library at a multiplicity of infection of 0.2, ensuring 500× coverage of the library. Selection of transduced cells commenced 24 h post transduction, utilizing 1.0 μg/mL puromycin for a period of 7 days, culminating in the establishment of a comprehensive genome knockout library cell line, designated MC38‐mBrie. Following 1 week of in vitro cultivation, both the MC38‐mBrie cells and the NTC cells were inoculated subcutaneously into the right rear groin of C57BL/6 mice at a density of 16 × 10^6^ cells per site. Tumor growth in the mice was closely monitored, with measurements taken every 3 days using vernier calipers. All tumor measurements were performed in a blinded manner. On Day 10 postinoculation, the mice were humanely euthanized, and the tumors were harvested for analysis. Simultaneously, an equivalent number of MC38‐mBrie cells were cultured in vitro for 10 days, serving as a control group for the screen process. Genomic DNA from all samples was extracted using the Universal Genomic DNA Purification Mini Spin Kit. The sgRNA libraries were read out through a two‐step PCR strategy. In the initial PCR (PCR#1), a region encompassing the sgRNA cassette was amplified with primers detailed in Table S5, ensuring sufficient genomic DNA was included to maintain the full library complexity. The second PCR (PCR#2) utilized the pooled PCR#1 products, employing barcoded secondary primers (as listed in Table S5) to incorporate the necessary sequencing adapters. The PCR#2 products were pooled and normalized for each biological replicate before merging the uniquely barcoded samples. The combined product was purified using the QiaQuick kit (QIAGEN) and quantified using a Nanodrop spectrometer. The libraries were subsequently diluted and sequenced on the NovaSeq. 6000 platform (Illumina).

### Data analysis of genome‐wide CRISPR‐Cas9 knockout screen

The sequencing quality was checked by fastqc (0.12.1) for the raw single‐end fq read files, and the reads were almost above Q35. The default parameters of fastp (0.24.0) software were applied to cut the sequencing adapters, remove low‐quality or duplicated reads. Since each fq file contains sequencing data from multiple samples, the seqkit tool suite was employed for alignment and data splitting. The 5' end of reads started with 8 bp sample barcode and 20 bp sgRNA spacer sequences. To avoid the potential problem of the sgRNA sequence matching the barcode during index construction, *seqkit subseq* command was used to split and extract the sequence before the sgRNA. The *seqkit grep ‐p barcode* command was used to match and extract the reads ID corresponding to sample barcodes, and then the fq file was split into each sample by the *seqkit grep ‐f ID.file* command according to the reads ID.

Raw single‐end fastq read files were filtered and demultiplexed using Cutadapt. The following settings: cutadapt ‐discard‐untrimmed ‐a GTTTTAGAGCTAGAAATGGC was used to remove extra sequences downstream of the sgRNA spacer sequences. As the forward PCR primers used to readout sgRNA representation were designed to have a variety of barcodes to facilitate multiplexed sequencing, we then demultiplexed these filtered reads with the following settings: cutadapt ‐g file: fbc.fasta–no‐trim, where fbc.fasta contained the possible barcode sequences within the forward primers. Finally, the following settings: cutadapt –discard‐untrimmed –g GTGGAAAGGACGAAACACCG was used to remove extra sequences upstream of the sgRNA spacers. Through this procedure, the raw fastq read files could be pared down to the 20 bp sgRNA spacer sequences.

Having extracted the 20 bp sgRNA spacer sequences from each demulitplexed sample, we then mapped the sgRNA spacers to the mBrie library (Table S1). To do so, we first generated a bowtie index of either sgRNA library using the bowtie‐build command in Bowtie1.1.2. Using these bowtie indexes, we mapped the filtered fastq read files using the following settings: bowtie‐v 1–suppress 4,5,6,7–chunkmbs 2000 –best. Using the resultant mapping output, we quantitated the number of reads that had mapped to each sgRNA within the library. For alignment specificity and noise control, only uniquely mapped reads (unique best hits) were retained; reads with equally good multiple alignments were discarded rather than randomly assigned. In barcode demultiplexing conflicts, ambiguous reads were also discarded. To generate sgRNA representation bar plots, we set a detection threshold of 10 reads, and counted the number of unique sgRNAs present in each sample.

The number of reads in each sample was normalized by converting raw sgRNA counts to reads per million (rpm). The rpm values were then subject to log_2_ transformation for certain analyses. To generate correlation heatmaps, we used the NMF R package and calculated the Pearson correlations between individual samples using log_2_ rpm counts. We first averaged the normalized sgRNA counts across all samples within a given group to calculate the cumulative distribution function for each sample group. To generate empirical cumulative distribution plots, we then used the ecdfplot function in the latticeExtra R package. Group‐wise distribution differences were statistically assessed using the two‐sample Kolmogorov–Smirnov test, with a statistically significant difference observed between the experimental and control groups (*p* < 0.05).

To enhance the robustness of hit identification, the enrichment of sgRNA and the corresponding gene were calculated with RNAi Gene Enrichment Ranking (RIGER) and the Model‐based Analysis of Genome‐wide CRISPR/Cas9 Knockout (MAGeCK) as described [57]. MAGeCK models sgRNA read counts using a negative binomial distribution and compares their enrichment against a null distribution derived from nontargeting controls, offering sensitivity in detecting subtle but consistent depletion signals. In parallel, RIGER assesses gene‐level enrichment based on the rank consistency of multiple sgRNAs per gene, providing robustness against potential off‐target effects of individual guides. For RIGER analysis, read count tables were used to calculate log_2_ fold changes for tumor versus cell samples using raw counts with a pseudo‐count of +1 per sgRNA to avoid infinities. Ties in rank were broken at random. This data was then used as input to a Java‐based implementation of RIGER (https://github.com/broadinstitute/rigerj) to generate gene rankings based on consistent enrichment across multiple sgRNAs for identification of candidate genes. Both the second highest‐ranking sgRNA and the weighted sum scoring methods were used for computation of gene rankings. Multiple‐testing correction was applied using the Benjamini–Hochberg procedure at both the sgRNA level (where applicable) and the gene level, and we report *q* values with a primary threshold of *q* < 0.05 (with sensitivity analysis at *q* < 0.10). For MAGeCK analysis, read count tables were used as inputs to a command‐line‐based tool (https://sourceforge.net/p/mageck/wiki/Home/) with the treatment group defined as the tumor samples and the control group defined as the cell pellet samples, with a list of nontargeting control sgRNAs provided for normalization and generation of the null distribution of RRA. Native MAGeCK plotting functions were used for visualization of RRA score and *p‐*value distributions and individual sgRNA read counts of selected genes. To generate Venn diagrams of overlapping enriched sgRNAs or genes, we considered all sgRNAs that were found to be significantly across RIGER and MAGeCK analyses.

### Subcellular localization and immunofluorescence assays

For subcellular localization assays, a transient expression vector, pCMV‐C9ORF50‐GFP was constructed. MC38 cells transiently transfected with pCMV‐C9ORF50‐GFP, were grown on glass coverslips. After 24 h, cells were fixed with 4% formaldehyde in PBS for 10 min at 4°C, followed by wash with PBS for three times. Coverslips were mounted on slides using anti‐fade mounting medium with DAPI. Cells were visualized with a Leica TCS SP8 imaging system in fluorescence imaging mode. 405 Diode laser was used for DAPI detection; 488 Argon was used for GFP detection.

For cell immunofluorescence, pCMV‐GFP and pCMV‐C9ORF50‐GFP transfected MC38‐sgNTC or MC38‐sgC9ORF50 cells were cultured in chamber slides overnight, and fixed with 4% formaldehyde in PBS for 10 min at 4°C, followed by permeabilization with 0.5% Triton X‐100 in PBS for 10 min, respectively. Cells were then blocked for nonspecific binding with 10% goat serum in PBS and 0.1% Tween‐20 (PBST) overnight, and incubated with the anti‐SF3B1 antibody (Abcam), coilin antibody (Abcam), SMN antibody (Abcam), U2AF65 antibody (Abcam), or unrelated IgG for 1 h at room temperature, followed by incubation with Alexa Fluor 568 or Alexa Fluor 647‐labeled secondary antibody (Invitrogen) for 30 min at room temperature. Coverslips were mounted on slides using anti‐fade mounting medium with DAPI. Immunofluorescence images were acquired on a Leica TCS SP8 imaging system in fluorescence imaging mode. 405 Diode laser was used for DAPI detection; 488 Argon was used for GFP detection; DPSS 561 was used for Alexa Fluor 568 detection.

### Protein recombination and purification

Recombinant proteins, including human C9ORF50‐GFP (hsaC9ORF50‐GFP) and mouse C9ORF50‐GFP (mmuC9ORF50‐GFP), were expressed in *E. coli* strain BL21‐CodonPlus® (DE3)‐RIPL (Agilent Technologies). Following induction, cells were harvested and lysed by sonication in an appropriate lysis buffer. The lysates were centrifuged, and the supernatants were subjected to initial purification using Ni‐NTA affinity chromatography (Sangon Biotech). The bound proteins were washed extensively with lysis buffer to remove nonspecific contaminants and subsequently eluted with elution buffer (20 mM Tris‐HCl, pH 7.5, 150 mM KCl, and 250 mM imidazole). The eluted proteins were concentrated using a centrifugal filtration device (30 kDa molecular weight cutoff) and further purified by size‐exclusion chromatography (SEC) on an SD2000 column (GE Healthcare). The purified proteins were buffer‐exchanged into storage buffer (20 mM HEPES, pH 7.4, 150 mM KCl, 1 mM DTT, and 5% glycerol), flash‐frozen in liquid nitrogen, and stored at −80°C for subsequent use.

### *In vitro* phase separation assay

The in vitro phase separation assay was conducted in storage buffer with specified protein concentrations, and PEG8000 was added to reach a final concentration of 10% (w/v). The phase separation assay was conducted on glass‐bottom dishes (NEST), sealed with a clear adhesive film to prevent evaporation, and observed using an OLYMPUS FV3000 confocal microscope with 60× oil immersion lenses. The phase separation assay using human or mouse C9ORF50 was performed in a physiological LLPS buffer (20 mM Tris‐HCl, pH 7.5, 300 mM NaCl, 130 mM KCl, 5 mM KH2PO4, 1.5 mM MgCl2, and 1 mg/mL BSA).

### Fluorescence recovery after photobleaching (FRAP)

FRAP were performed procedures as previously described [58]. FRAP experiments were performed on an OLYMPUS FV3000 confocal microscope with a 60× oil immersion objective at 37°C in a live‐cell‐imaging chamber. droplets were bleached with a 488‐nm laser pulse (60% intensity, 0.5 s). The recovery from photobleaching was recorded for the indicated time. The pre‐bleach signal was normalized to 100%. Analysis of the recovery curves was carried out with FIJI/ImageJ software.

### Co‐immunoprecipitation assay and mass spectrometry

MC38 or HEK293T cells were transfected with 10 μg of either the GFP‐tagged *C9ORF50* expression vector or a GFP‐tag control plasmid. Protein harvesting occurred 48 h posttransfection, with extraction carried out using RIPA lysis buffer supplemented with a protease inhibitor cocktail (Roche). After a 10‐min incubation on ice, the lysates were analyzed for protein concentration via the BCA method. Co‐immunoprecipitation was performed using the Pierce Classic Magnetic IP/Co‐IP kit (Thermofisher) according to manufacturer's instructions. A total of 2 mg of protein was subjected to immunoprecipitation with 10 μg of rabbit anti‐GFP antibody. The immunoprecipitated complexes were then separated on SDS‐PAGE followed by Coomassie Blue staining. Gel fragments were excised, detained in 50% ethanol and 5% acetic acid, dehydrated in acetonitrile, dried in a Speed vacuum, and digested with trypsin. Tryptic peptides were separated on a C18 column and analyzed by LTQ‐Orbitrap Velos (Thermo). The peptides were extracted from the polyacrylamide and subjected to liquid chromatography‐mass spectrometry (MS) analysis. Protein quantification was performed using MaxQuant against the UniProt human database (homo_sapiens_uniprot_2023_3_13). For the interactome analysis, proteins were considered specific interactors only if they exhibited a ≥2‐fold enrichment in *C9ORF50* immunoprecipitates compared to nontarget control (NTC) samples, with the identified proteins in each group first filtered using corresponding IgG control. Additionally, statistical significance was determined with a threshold of *p* < 0.05, utilizing the student's *t*‐test with Benjamini–Hochberg correction for multiple testing. The proteins identified through mass spectrometry were further validated by co‐immunoprecipitation and Western blotting analysis.

### Western blot

For protein extraction from cultured cells, MC38‐sgC9ORF50 and MC38‐sgNTC cells were lysed using RIPA buffer supplemented with 1 mM Na₃VO₄, 1 μg/mL leupeptin, and 0.5 mM PMSF, and incubated on ice for 30 min. For protein extraction from tissue samples, approximately 40 mg of tissue was washed with ice‐cold PBS, minced into small fragments, and ground into a fine powder under liquid nitrogen. The powder was homogenized in 500 μL of RIPA buffer supplemented with the same inhibitors as above (1 mM Na₃VO₄, 1 μg/mL leupeptin, and 0.5 mM PMSF), followed by lysis on ice for 30 min. The lysates were then centrifuged, and the supernatant was collected for protein concentration determination using a bicinchoninic acid (BCA) assay. For Western blot analysis, 20 μg of each protein sample was loaded and separated via SDS‐PAGE, then transferred onto PVDF membranes (Merck Chemicals). Following membrane blocking, primary antibodies were applied overnight at the indicated concentrations in a 2% BSA‐TBST solution: anti‐Phospho‐IRF3 (Ser396), anti‐IRF3, anti‐Phospho‐TBK1 (Ser172), anti‐TBK1, anti‐SDHA, anti‐GFP, anti‐U2SURP (all from ThermoFisher), anti‐SRSF12 (from Aifang biological). The sources and product information for all antibodies used in this study are provided in Table S6. Subsequently, the membranes were incubated with secondary antibodies, goat anti‐rabbit HRP and rabbit anti‐mouse HRP (both from ThermoFisher), for 1 h. Detection was performed using the SuperSignal™ West Pico PLUS chemiluminescent substrate (ThermoFisher).

### RNA‐seq analysis

Total RNA was extracted from MC38‐sgC9ORF50 and MC38‐sgNTC cells using TRIzol reagent (Invitrogen), following the manufacturer's instructions. Thereafter, the NEBNext Ultra RNA Library Prep Kit for Illumina (New England Biolabs) was employed to construct the sequencing libraries. The mRNA from total RNA was enriched using Oligo‐dT beads and reverse‐transcribed into double‐stranded cDNA, sample clustering was performed using the TruSeq PE Cluster Kit v3‐cBot‐HS (Illumina), and sequencing was conducted on the Illumina NovaSeq. 6000 platform. Differential expression analysis was conducted on three biological replicates per group, utilizing the DESeq2 R package (version 1.16.1). DESeq2 provided statistical routines for determining differential expression in digital gene expression data, employing a model based on the negative binomial distribution. The analysis was performed using raw count data without prior normalization [59]. DESeq2's shrinkage estimator was specifically employed for log_2_ fold change calculation, which reduces the magnitude of fold changes for genes with high variability or low counts. The resulting *p*‐value was adjusted using the Benjamini and Hochberg approach for controlling the FDR. Genes with an adjusted *p* < 0.05 and |log_2_FC| > 1, as determined by DESeq2, were assigned as differentially expressed. The differential expression analysis results were visualized using volcano plots.

Enrichment analysis was conducted using the clusterProfiler package (version 3.18.1) in R. The DEGs were subjected to Gene Ontology (GO) functional enrichment analysis, KEGG pathway enrichment analysis, and GSEA. For GO analysis, the biological process (BP), molecular function (MF), and cellular component (CC) categories were examined. KEGG pathway analysis was performed to identify significantly enriched biological pathways. For GSEA, gene sets were pre‐ranked based on their log2 fold changes, and enrichment was calculated using default parameters. All enrichment analyses were considered statistically significant at an adjusted *p*‐value < 0.05 [60].

### Quantitative PCR assay

Total RNA was extracted from sgC9ORF50 and sgNTC MC38 cells using TRIzol reagent (Invitrogen) and subsequently reverse‐transcribed into complementary DNA (cDNA) with SuperScript III Reverse Transcriptase (Invitrogen), following the manufacturer's protocol. Quantitative real‐time PCR (qPCR) was conducted in 96‐well plates using the SYBR Green PCR Master Mix (Roche). The fluorescence intensity was measured using a Light Cycler 480 instrument (Roche). Prime sequences for amplification are detailed in Table S5.

### Detection and quantitation of cytokines and chemokines

MC38‐sgC9ORF50 and MC38‐sgNTC cells were cultured in standard media for 48 h. Supernatants and whole‐cell extracts were collected, with the latter obtained either through the freeze‐thaw lysis method or using RIPA buffer. To prevent protein degradation, a protease inhibitor cocktail was incorporated into all samples. The concentrations of cytokines, including IFNα, IFNβ, IL‐6, and TNF‐α, as well as chemokines including CXCL9, CXCL10, and CXCL11, were quantified using specific enzyme‐linked immunosorbent assay (ELISA) kits (Mouse IL‐6 ELISA Kit, Mouse IFN‐α ELISA Kit, Mouse IFN‐β ELISA Kit, Mouse TNF‐α ELISA Kit, Mouse CXCL9 ELISA Kit, Mouse CXCL10 ELISA Kit, and Mouse CXCL11 ELISA Kit).

### ATAC‐seq analysis

ATAC‐seq libraries were prepared following established protocols [61]. Cell populations of MC38‐sgC9ORF50 and MC38‐sgNTC, each at a density of 1 × 10^4^ cells, were lysed, and accessible chromatin was tagged by transposition with the Nextera Tn5 transposase. The resulting transposed DNA fragments were purified using the Qiagen MinElute kit and subjected to amplification for 5–10 cycles with Nextera PCR primers. The ATAC‐seq libraries were then submitted for paired‐end sequencing on an Illumina NovaSeq. 6000 platform. The raw FASTQ reads obtained were mapped to the mm10 genome assembly using Bowtie v2, and accessible genomic regions were identified with MACS2. These accessible regions across all samples were consolidated, and the read counts within each region were tallied. Pairwise Spearman correlation coefficients were calculated based on the read counts within each region to assess the reproducibility and similarity between samples. Differential accessibility analysis was conducted using DESeq2, which evaluates statistical significance in chromatin accessibility between conditions. The intersection of accessible regions with motif analysis was performed using HOMER, providing insights into the transcription factor binding events associated with these regions. Promoter regions were defined as ±2 kb from the transcription start site (TSS) of the longest transcript for each gene.

### RNA immunoprecipitation

RIP was conducted as previously reported [62]. MC38‐sgNTC and MC38‐sgC9ORF50 cells were collected and lysed in buffer containing 50 mM Tris, pH 7.5, 150 mM NaCl, 1 mM EDTA, and 1% TritonX supplemented with cOmplete protease inhibitor (Roche) and SUPERase In (Invitrogen). Lysate was then incubated with J2 dsRNA‐specific antibody (Cell Signaling Technology) or control IgG (Cell Signaling Technology). RNA‐protein complexes were immunoprecipitated with protein A agarose beads and RNA was isolated by adding TRIzol to the beads and glycogen (Thermo Fisher Scientific) added as a carrier to aid the precipitation reaction. 1% of total lysate was saved for input. Extracted RNA was reverse‐transcribed using Advantage RT‐for PCR kit (Clontech) with oligo dT and subsequently qPCR was performed. %Input = 2^−∆Ct^ × 100%; ∆Ct = Ct_RIP_ – [Ct_input_ – dilution factor]. Sequences of primers are listed in Table S5. Additionally, in MC38 cells, overexpression was performed using pCMV‐GFP and pCMV‐C9ORF50‐GFP plasmids respectively, followed by RIP‐qPCR experiments using GFP Monoclonal Antibody (N86/8) following the same experimental procedures as described above.

### Flow cytometry and cell sorting

Both sgC9ORF50 and sgNTC‐transduced MC38 cells were inoculated subcutaneously into the right flanks of C57BL/6 or nude mice, respectively, at a density of 2 × 10^6^ cells per site. Tumor tissues were harvested, mechanical minced (200 μm mesh), and treated with a digestion solution comprising PBS with 2% FBS, collagenase type IV (0.25 mg/mL, Sigma), and DNase I (20 U/mL, Sigma) for 45 min. The mixture was then agitated on a shaker incubator at 37°C for 10 min to facilitate disaggregation, followed by vigorous pipetting with a 10‐mL pipette. The resulting cell suspension was strained through a 70‐μm nylon mesh to obtain a single‐cell suspension. The single‐cell suspensions were incubated with a rat anti‐mouse CD16/CD32 monoclonal antibody (ThermoFisher) to block Fc receptor‐mediated reactions, followed by the surface staining at 4°C for 15 min. A panel of antibodies (BioLegend) was used for surface staining: APC‐CD45, PE‐cy7‐CD11b, percp/cy5.5‐MHC‐II IA/IE, eFluor450‐Ly6c, PE‐F4/80, PE/Dazzle‐CD11c, eFluor506‐L/D, and FITC‐NKP46, FITC‐H2‐ H‐2K^b^ [63]. For detection of T‐bet expression, surface‐labeled cells were fixed and permeabilized using the Transcription Factor Buffer Set (BD Biosciences) following the manufacturer's protocol, followed by intracellular staining with APC‐T‐bet antibody for 40–50 min at 4°C. To assess IFN‐γ production, cells were collected, washed, fixed, and permeabilized with the Fixation/Permeabilization Solution Kit (BD Biosciences), then incubated with a FITC‐conjugated anti‐IFN‐γ antibody. Appropriate fluorescein‐conjugated isotype control antibodies were included in all experiments as negative controls. The samples were analyzed using a fluorescent‐activated cell sorter (FACS) Aria III cell sorter (BD Biosciences) with BD FACSDiva Software (v9.0, BD Biosciences). Data analysis was conducted using FlowJo software (version 10.8).

### Cell apoptosis and proliferation analyses

Cell apoptosis was measured by Annexin V‐FITC/propidium iodide (PI) assay using flow cytometry as previously described. Specifically, cells were subjected to staining with the Annexin V‐FITC/PI apoptosis detection kit (Yeasen) and subsequently analyzed using a FACS Aria III cell sorter (BD Biosciences). In the cell proliferation assay, 5‐ethynyl‐2'‐deoxyuridine (EdU) was introduced to the culture medium at a final concentration of 1 μM, added 2 h before detection and maintained until cell collection. At the designated time points, cells were trypsinized and processed for the Click‐iT EdU assay following the manufacturer's instructions (ThermoFisher). The proliferating cells were then quantified using the FACS Aria III cell sorter (BD Biosciences). Tumor cell apoptosis was assessed by TUNEL staining, and proliferation was evaluated via Ki67 immunostaining. Nuclei were counterstained with DAPI.

Caspase‐3 and Caspase‐8 activity was assessed in MC38‐sgNTC and MC38‐sgC9ORF50 cell lines using a colorimetric assay. Briefly, cells were collected, digested, and lysed on ice. The cell lysates were centrifuged, and the supernatants were harvested (with protein concentration determined by the Bradford assay afterward). For the enzyme activity assay, reaction mixtures were prepared by adding specific colorimetric substrates (Ac‐DEVD‐pNA for Caspase‐3, Ac‐IETD‐pNA for Caspase‐8) to the lysates along with detection buffer, then incubated at 37°C. After incubation, the absorbance at 405 nm (A₄₀₅) was measured using a microplate reader. A standard curve of p‐nitroaniline (pNA) concentration versus A₄₀₅ was generated (R² = 0.9992) to quantify the pNA released by Caspase‐3/‐8‐mediated substrate cleavage. Caspase‐3/‐8 activity was defined as the amount of enzyme cleaving 1.0 nmol of the colorimetric substrate per hour at 37°C under saturated substrate conditions, and normalized to the total protein concentration of the lysates.

### Tissue H&E and immunofluorescence staining

For H&E staining, paraffin‐embedded tumor tissue sections were deparaffinized in xylene and subjected to a graded ethanol series for rehydration. Antigen retrieval was performed using heat in citrate buffer. Subsequently, the tumor tissue sections were stained with hematoxylin for 1 min, followed by a brief rinse and eosin staining for 1 min. After additional rinsing, the slides were mounted with neutral balsam for preservation. For immunofluorescence, sections were also deparaffinized, rehydrated and subjected to antigen retrieval. The slides were then blocked with 5% goat serum in phosphate‐buffered saline (PBS), and subsequently incubated with primary antibodies specific for CD3 (ThermoFisher), CD4 (ThermoFisher), and CD8 (ThermoFisher), J2 dsRNA‐specific antibody (Cell Signaling Technology) alongside a rabbit IgG isotype control (ThermoFisher). A TSA indirect kit (PerkinElmer) was used according to the manufacturer's instructions. Imaging was conducted using the Vectra® Polaris™ Imaging System (Akoya Biosciences), and subsequent image analysis was performed utilizing the HALO™ Image Analysis Software.

### scRNA‐seq analysis

Tumor tissues from the MC38‐NTC (*n* = 3) and MC38‐sgC9ORF50 (*n* = 3) models were harvested from tumor‐bearing mice to prepare single‐cell suspensions. Tumor‐infiltrating leukocytes were enriched through FACS, specifically isolating CD45^+^ cells to focus on immune cell populations. The cell suspensions were then counted and loaded onto a 10× Genomics Chromium platform for single‐cell RNA sequencing, following the manufacturer's protocol.

The analysis of the generated sequencing data was performed using Cell Ranger (version 4.0.0), which facilitated sample demultiplexing, barcode processing, alignment to the mouse genome (GRCm38), filtering of low‐quality reads, counting of unique molecular identifiers (UMIs), and aggregation of sequencing runs. The resulting UMI count matrix was subsequently processed using Seurat (version 4.3.0) for downstream analyses. Low‐quality cells were filtered based on the following criteria: a minimum of 200 detected genes and an upper limit of 7000 detected genes. Additionally, cells with more than 30% mitochondrial UMI counts or more than 0.3% hemoglobin UMI counts were excluded. Normalization and variance stabilization of the count data using the SCTransform function in Seurat. For data integration across different experimental groups, we employed the SCT integration method. The constructed shared nearest neighbor (SNN) graph was generated using the FindNeighbors function, and clustering was executed with the FindClusters function, setting the resolution parameter to 0.5. To visualize the clustered data further, we implemented UMAP and t‐SNE for nonlinear dimensionality reduction. UMAP was performed using the first 30 PCA dimensions, while t‐SNE was executed on the same set of dimensions, with results plotted using the DimPlot function. The FindAllMarkers function in Seurat was utilized to identify differentially expressed features that characterize each cluster.

For cell annotation, we employed both database‐based and marker‐based approaches. The database‐based auto‐annotation utilized the SingleR package along with ImmGenData and MouseRNAseqData reference datasets. In the marker‐based auto‐annotation, we prepared a list of specific marker genes corresponding to various cell lineages, including lymphoid, myeloid, epithelial, and stromal cells. Leveraging the results from these different annotation methods, clusters were assigned to specific cell types based on the expression of key marker genes. For two‐group comparisons (e.g., NTC vs sgC9ORF50) of gene expression and module scores, we used the non‐parametric Wilcoxon rank‐sum (Mann–Whitney) test, with BH‐adjusted *p* values reported. All tests were two‐sided unless specified, and sensitivity analyses (e.g., *q* < 0.10, alternative post hoc corrections) yielded consistent conclusions.

### RNA interference assay

Small interfering RNAs (siRNAs) targeting *C9orf50*, as well as control scramble siRNAs, were designed and synthesized by GenePharma (Table S7). *RIG‐I* and *MDA5* siRNA sequences (two independent duplexes per gene) were purchased from Santa Cruz Biotechnology, Inc. (Santa Cruz) [64, 65]. To enhance intracellular stability, cholesterol‐modified version of the C9ORF50‐siRNAs (M‐siC9ORF50‐1 and M‐siC9ORF50‐2) and a corresponding cholesterol‐modified scramble siRNA (M‐siScramble) were also produced. For functional assays, MC38‐sgC9ORF50 cells received siRig‐I‐1/2 or siMda5‐1/2 with scrambled controls, while wild‐type MC38 cells were transfected with siC9ORF50‐1/2 or scrambled controls. Transfections employed Lipofectamine™ RNAiMAX (Invitrogen) following manufacturer protocols, with cells harvested at 48 h posttransfection. siRNA efficacy (>70% mRNA knockdown) and specificity were validated by qPCR, selecting optimal duplexes for downstream experiments. Complete sequences are documented in Table S5.

Wild‐type MC38 cells were injected subcutaneously into C57BL/6 mice. On the sixth day following tumor transplantation, mice were randomized into groups (*n* = 5 per group) to receive either scramble siRNA (siScramble), siC9ORF50, M‐siC9ORF50‐1, and M‐siC9ORF50‐2), or M‐siScramble) by intratumoral injection. Dose calculation follows 1 mg/kg based on animal weight at injection [66]. The injection formulation contained 30 μg of siRNA per mouse, administered every 2 days for a total of three injections. For the combination therapy, an anti‐PD1 antibody and a rat IgG2a isotype‐matched control were started at the same time and were given intraperitoneally at a dose of 200 μg per mouse every 3 days for a total for four doses. Throughout the treatment period, the tumor‐bearing mice were closely observed, and tumor dimensions were measured every 3 days using vernier calipers in a blinded manner relative to cage labels. Following euthanasia, the tumors were harvested, weighed, and subjected to H&E staining as well as immunofluorescence analysis.

### Protein structure prediction and functional annotation

The sequence of C9ORF50 and its homologs was obtained from the UniProt database (https://www.uniprot.org/). Multiple sequence alignments were performed using the MUSCLE algorithm (https://toolkit.tuebingen.mpg.de/tools/muscle) and visualized with the R package ggmsa and Adobe Illustrator. The sequence of C9ORF50 was submitted to ColabFold (AlphaFold2) for structure prediction. Additionally, the AlphaFold3 web server (https://alphafoldserver.com/) was used for further structural analysis. Computational disorder profiling was performed using IUPred2, ANCHOR2, and MetaPredict algorithms. IDRs were defined by consensus across at least two algorithms with residue probability thresholds >0.5, visualized through MobiDB's integrated platform. Sequence composition analysis calculated order‐promoting residues (OPR: Trp, Cys, Tyr, Ile, Phe, Val, Asn, Leu) and disorder‐promoting residues (DPR: Arg, Pro, Gln, Gly, Glu, Ser, Ala, Lys) based on established biochemical classifications. Percentages were determined for human and mouse C9ORF50, with canonical IDPs (α‐synuclein, osteopontin, emerin, TFIP11) as reference standards. Charge‐Hydropathy (CH) analysis was conducted using the PONDR® v.2007 platform implementing Uversky's established methodology [67, 68].

### Immunoblot analysis of ubiquitinated proteins

sgC9ORF50 and sgNTC control cells were seeded in 10‐cm dishes and cultured for 24 h. Where indicated, proteasomal inhibition was carried out by treating the cells with 15 μmol/L MG132 for 3 h before harvest; control groups received an equivalent volume of DMSO alone. After treatment, the cells were washed twice with ice‐cold PBS and lysed with RIPA buffer supplemented with protease inhibitors. The lysates were centrifuged at 12,000 × *g* for 15 min at 4°C, and the resulting supernatant was collected. Protein concentration was measured using a BCA assay (Abcam). Equal amounts of protein (20 μg per lane) were separated by SDS‐PAGE and transferred to PVDF membranes. The membranes were immunoblotted with an anti‐ubiquitin monoclonal antibody (Thermo Fisher Scientific). To verify equal loading, the blots were reprobed with an anti‐SDHA antibody. Quantification of ubiquitinated proteins was performed by grayscale densitometric analysis using ImageJ, with values normalized to SDHA signals.

### ELISpot assay

IFN‐γ ELISpot assays were performed using mouse‐specific kits (BD Biosciences) in accordance with the manufacturer's protocol. Briefly, 96‐well filtration plates were coated with an IFN‐γ capture monoclonal antibody and left overnight at 4°C. After washing, the plates were blocked with RPMI‐1640 medium supplemented with 10% FBS for 2 h at room temperature. Tumor‐infiltrating immune cells (TIICs) isolated from the different treatment groups (M‐siC9ORF50‐1, M‐siC9ORF50‐2, or M‐siScramble) were counted and resuspended in complete RPMI‐1640 medium with 10% FBS. A total of 1 × 10⁴ TIICs per well were seeded into the pretreated plates and cultured for approximately 48 h at 37°C under 5% CO₂. Subsequently, biotinylated detection antibody was added and incubated for 2 h at room temperature. Following three washes with PBST, the plates were treated with horseradish peroxidase (HRP)‐conjugated streptavidin for 1 h at room temperature. Spot development was carried out using an AEC substrate reagent kit (BD Biosciences), and the resulting spots were quantified with an Immunospot Reader (Cellular Technology).

### Analysis of public genomic datasets

TCGA pan‐cancer gene expression and clinical data were obtained from the UCSC Xena platform (https://xenabrowser.net/). Expression data of *C9ORF50* were extracted and integrated with corresponding clinical information across multiple cancer types. Only samples with complete survival data (progression‐free interval status and time) and detectable *C9ORF50* expression (expression values > 0) were included in the analysis. PFI time was converted to years for visualization purposes. Event status was determined based on documented progression or death (event = 1) versus no progression (event = 0). Statistical analyses were performed using R (version 4.4) with the “survival” and “survminer” packages [69]. For each cancer type, optimal *C9ORF50* expression cutpoints were determined using the maximally selected rank statistics implemented in the surv_cutpoint function from the “survminer” package. This approach identifies the expression threshold that provides the most significant split between high and low‐expression groups in terms of survival outcomes. Patients were stratified into “high” and “low” *C9ORF50* expression groups based on these optimal cutpoints. Kaplan–Meier survival curves were generated using the surv_fit function, and differences in survival probabilities between groups were assessed using the log‐rank test. Median survival times were calculated when applicable and indicated with horizontal and vertical lines on the survival curves. Survival curves were visualized using the ggsurvplot function with 95% confidence intervals. Risk tables were optionally included to show the number of patients at risk at various time points. All plots were generated using the “ggplot2” package. *p* values less than 0.05 were considered statistically significant and displayed on each plot. For specific cancer types of interest (e.g., COAD/READ), additional analyses were performed to assess *C9ORF50* expression across different pathological stages. Expression differences between stages were visualized using violin plots with overlaid boxplots and jittered points, and statistical comparisons were conducted using the Kruskal–Wallis test followed by pairwise Wilcoxon rank‐sum tests with Benjamini–Hochberg correction for multiple comparisons. Spearman correlation analysis was performed to evaluate associations between *C9ORF50* mRNA expression and immune parameters. Immune cell abundances were inferred using established deconvolution algorithms (e.g., TIMER, CIBERSORT). For volcano plots, immune cells or chemokines significantly correlated with *C9ORF50* expression (adjusted *p* < 0.05, Benjamini–Hochberg method) were highlighted in green (negative correlation) or red (positive correlation). Analyses were carried out in R using stats and ggplot2 packages.

### Human tissue samples

Colorectal cancer and paired normal tissue samples (located > 5 cm from the malignant region) were obtained during surgery. All patients underwent resection of the primary tumor at the Second Xiangya Hospital of Central South University. Informed consent was obtained from all participants (Table S8). We have complied with all relevant ethical regulations for work with human participants.

### Statistics and reproducibility

Data between two groups were analyzed using a two‐tailed unpaired *t*‐test. For longitudinal comparisons (e.g., tumor growth curves), a two‐way ANOVA with Holm–Sidak's multiple comparisons test was applied to assess the effects of group (e.g., sgC9ORF50 vs. sgNTC) and time, including their interaction term. One‐way ANOVA with Tukey's post hoc test was performed for multi‐group comparisons. Different levels of statistical significance were assessed based on specific *p* values and type I error cutoffs (0.05, 0.01, 0.001). GraphPad Prism (version 10.1.2) Software and RStudio (version 2022.12.0) were used for these analyses.

## AUTHOR CONTRIBUTIONS

**Tong Shao**: Methodology; data curation; validation; investigation; funding acquisition; writing—original draft; software; formal analysis; visualization. **Chuanyang Liu**: Methodology; Software; investigation; writing—original draft; data curation; formal analysis; visualization. **Jingyu Kuang**: Methodology; validation; investigation; visualization; writing—original draft. **Sisi Xie**: Methodology; data curation; investigation; validation. **Ying Qu**: Software; data curation. **Yingying Li**: Investigation; validation; formal analysis; visualization. **Lulu Zhang**: Data curation; investigation; validation; formal analysis. **Fangzhou Liu**: Investigation; validation; formal analysis; data curation. **Yanhua Qi**: Software; Data curation. **Tao Hou**: Data curation; Investigation; Resources. **Ming Li**: Software; data curation. **Sujuan Zhang**: Investigation; validation. **Yu Liu**: Investigation; validation. **Zhixiang Yuan**: Investigation; validation. **Jiali Liu**: Investigation; validation. **Yanming Hu**: Investigation; validation. **Jingyang Wang**: Investigation; validation. **Chenghu Song**: Investigation; validation. **Shaowei Zhang**: Investigation; validation. **Lingyun Zhu**: Investigation; validation. **Jianzhong Shao**: Investigation; validation. **Aifu Lin**: Writing—review and editing; supervision. **Wenjun Mao**: Supervision; writing—review and editing. **Guangchuan Wang**: Writing—review and editing; project administration. **Lvyun Zhu**: Funding acquisition; writing—review and editing; supervision; project administration; conceptualization. All authors have read the final manuscript and approved it for publication.

## CONFLICT OF INTEREST STATEMENT

The authors declare no conflicts of interest.

## ETHICS STATEMENT

The ethics application of animal studies (No. 2023050) was approved by the Animal Welfare and Ethics Committee of Changsha Medical University. The ethics application of human clinical samples (No. Z0102) was approved by the Ethics Committee of the Second Xiangya Hospital of Central South University.

## Supporting information


**Figure S1:** Additional analyses for genome‐scale in vivo CRISPR screen.
**Figure S2:** Validation of the convergent top hits from genome‐scale in vivo CRISPR screen.
**Figure S3:** *C9ORF50* knockout suppresses cancer progression.
**Figure S4:** Structural analysis of C9ORF50.
**Figure S5:** C9ORF50 interacts with spliceosome components.
**Figure S6:** Transcriptomic sequencing and data analysis.
**Figure S7:** Transcriptome analysis of RNA splicing process influenced by *C9ORF50* knockout.
**Figure S8:** *C9ORF50* deficiency activates innate immunity through dsRNA sensing without affecting antigen presentation or global proteostasis.
**Figure S9:** Additional analysis of tumor immune infiltration profile influenced by *C9ORF50* knockout.
**Figure S10:** *C9ORF50* knockout induces immune cell state reprogramming in the tumor microenvironment.
**Figure S11:** *C9ORF50* deficiency leads to production of chemokines.
**Figure S12:** *C9ORF50* deficiency correlates with enhanced anti‐tumor immunity and improved survival in colorectal cancer.
**Figure S13:** Additional analysis of cancer therapeutic effect of RNA interference targeting *C9ORF50*.

**Table S1:** Normalized reads of mBrie screen.
**Table S2:** Hits in MAGeCK.
**Table S3:** Hits in RIGER.
**Table S4:** Spliceosome‐related genes.
**Table S5:** Primers used in this work.
**Table S6:** Reagents used in the study.
**Table S7:** siRNA sequence.
**Table S8:** Clinical information of patient samples.

## Data Availability

The data that support the findings of this study are openly available in C9ORF50 at https://ngdc.cncb.ac.cn/, reference number PRJCA029612. All the sequencing data have been deposited in the National Genomics Data Center (NGDC) at the China National Center for Bioinformation (CNCB) under BioProject accession number PRJCA029612 (https://ngdc.cncb.ac.cn/bioproject/browse/PRJCA029612). TCGA pan‐cancer gene expression and clinical data were obtained from the UCSC Xena platform (https://xenabrowser.net/). The data and scripts used are saved in GitHub https://github.com/Travis13197/C9orf50_iMeta. Supplementary materials (figures, tables, graphical abstract, slides, videos, Chinese translated version, and update materials) may be found in the online DOI or iMeta Science http://www.imeta.science/.
