# Supplementary material for: Genome‐wide CRISPR screen reveals an uncharacterized spliceosome regulator as new candidate immunotherapy target
Source: Imeta. 2025 Nov 28;4(6):e70096. doi: 10.1002/imt2.70096 (PMC12747536; doi:10.1002/imt2.70096)
Supplement: Supplementary file 1 — Figure S1: Additional analyses for genome‐scale in vivo CRISPR screen. Figure S2: Validation of the convergent top hits from genome‐scale in vivo CRISPR screen. Figure S3: C9ORF50 knockout suppresses cancer progression. Figure S4: Structural analysis of C9ORF50. Figure S5: C9ORF50 interacts with spliceosome components. Figure S6: Transcriptomic sequencing and data analysis. Figure S7: Transcriptome analysis of RNA splicing process influenced by C9ORF50 knockout. Figure S8: C9ORF50 deficiency activates innate immunity through dsRNA sensing without affecting antigen presentation or global proteostasis. Figure S9: Additional analysis of tumor immune infiltration profile influenced by C9ORF50 knockout. Figure S10: C9ORF50 knockout induces immune cell state reprogramming in the tumor microenvironment. Figure S11: C9ORF50 deficiency leads to production of chemokines. Figure S12: C9ORF50 deficiency correlates with enhanced anti‐tumor immunity and improved survival in colorectal cancer. Figure S13: Additional analysis of cancer therapeutic effect of RNA interference targeting C9ORF50. [file IMT2-4-e70096-s001.docx]

**Supporting information to**

**Genome-wide CRISPR screen reveals an uncharacterized spliceosome regulator as a candidate immunotherapy target**

**Running title**: Splicing regulator C9ORF50 controls immune evasion and immunotherapy response

Tong Shao^1#^, Chuanyang Liu^1#^, Jingyu Kuang^1#^, Sisi Xie^1#^, Ying Qu^1^, Yingying Li^1^, Lulu Zhang^2^, Fangzhou Liu^3^, Yanhua Qi^1^, Tao Hou^4^, Ming Li^1,5^, Sujuan Zhang^4^, Yu Liu^1^, Zhixiang Yuan^1^, Jiali Liu^1^, Yanming Hu^4^, Jingyang Wang^1^, Chenghu Song^6,7^, Shaowei Zhang^1^, Lingyun Zhu^1^, Jianzhong Shao^3^, Aifu Lin^3^*, Wenjun Mao^6, 7^* Guangchuan Wang^2^*, Lvyun Zhu^1^*

^1^ College of Science, National University of Defense Technology, Changsha 410073, China

^2^ Key Laboratory of Multi-Cell Systems, Shanghai Institute of Biochemistry and Cell Biology, Center for Excellence in Molecular Cell Science, Chinese Academy of Sciences, Shanghai 20031, China

^3^ College of Life Science, Zhejiang University, Hangzhou 310058, China

^4^ Department of Oncology, The Second Xiangya Hospital, Central South University, Changsha 410012, China

^5^ Jiuquan Satellite Launch Centre, Jiuquan 73500, China

^6^ Department of Cardiothoracic Surgery, the Affiliated Wuxi People’s Hospital of Nanjing Medical University, Wuxi People’s Hospital, Wuxi Medical Center, Nanjing Medical University, Wuxi 214023, China.

^7^ Wuxi College of Clinical Medicine, Nanjing Medical University, Wuxi 214023, China

^#^These authors contributed equally: Tong Shao, Chuanyang Liu, Jingyu Kuang, Sisi Xie

*Correspondence: [zhulvyun@nudt.edu.cn](mailto:zhulvyun@nudt.edu.cn) (Lvyun Zhu), [guangchuan.wang@sibcb.ac.cn](mailto:guangchuan.wang@sibcb.ac.cn) (Guangchuan Wang), [maowenjun1@njmu.edu.cn](mailto:maowenjun1@njmu.edu.cn) (Wenjun Mao), [linaifu@zju.edu.cn](mailto:linaifu@zju.edu.cn) (Aifu Lin)

**Supplementary figure**

**
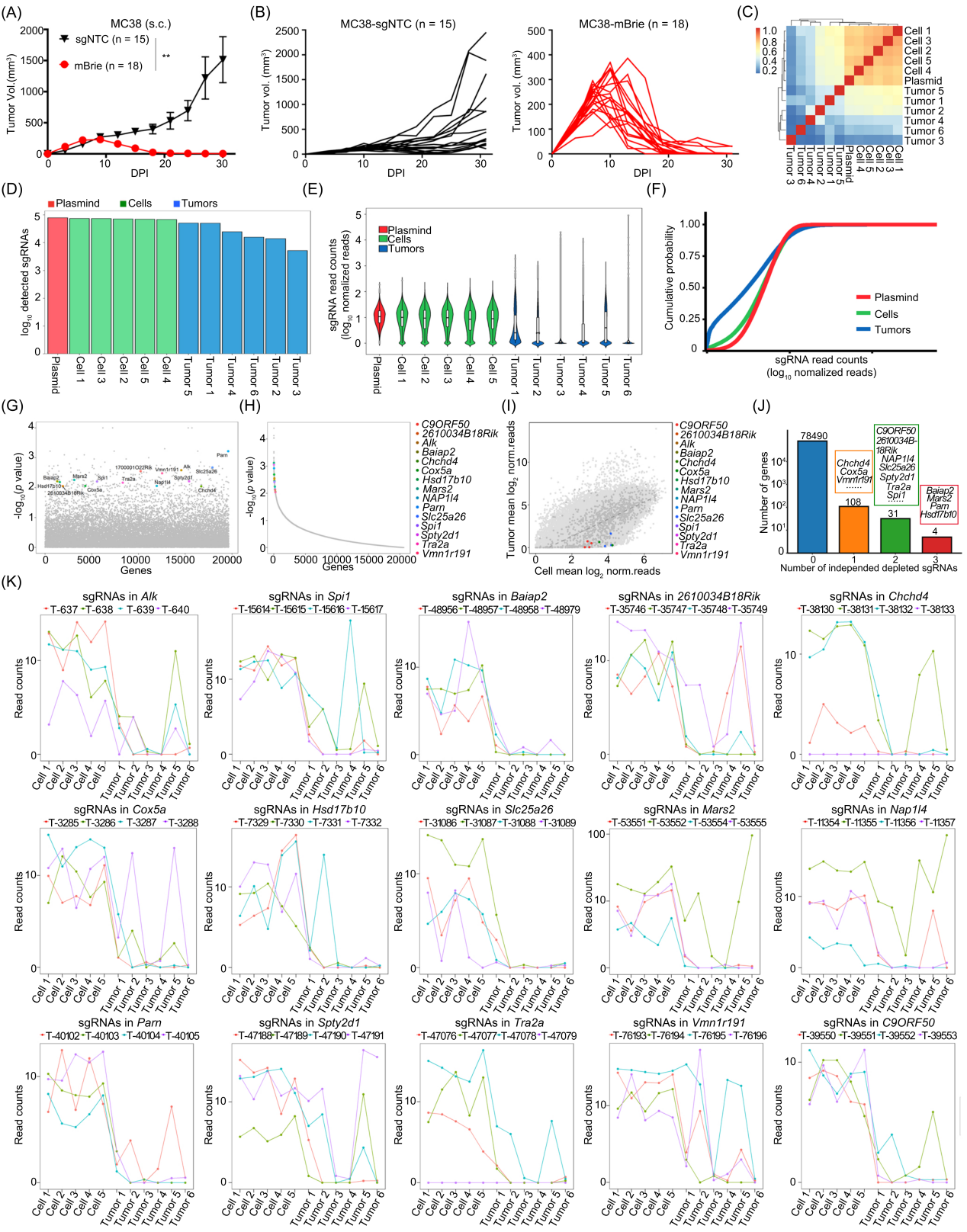
**

**Figure S1 Additional analyses for genome-scale *in vivo* clustered regularly interspaced short palindromic repeats (CRISPR) screen.** (A and B) Tumor growth curves (A) and individual tumor growth curves (B) of subcutaneously transplanted MC38 cells transduced with the mBrie library (n = 18 mice) or NTC sgRNA (n = 15 mice) in C57BL/6 mice. ***p* < 0.01 by two-way ANOVA with Holm-Sidak’s multiple comparisons test. Data are shown as mean ± SEM. (C) A heatmap showing the Pearson correlation coefficient of sgRNA library representations across all samples from the mBrie screen, including plasmid library (n = 1), library transduced cells (n = 5 biological replicates) before transplantation (day 7 post infection), and subcutaneous tumors in host (n = 6 biological replicates, 9 days post transplantation). (D) A bar plot showing number of detected sgRNAs in each sample. (E) A Violin Chart showing the sgRNA library representations across all samples from the mBrie screen. GTSs are shown in color, while NTCs are shown in grey. (F) A cumulative distribution function (CDF) plot showing the sgRNA library representations across all three sample types from the mBrie screen. Group-wise distribution differences were statistically assessed using the two-sample Kolmogorov–Smirnov test, with a statistically significant difference observed between the experimental and control groups (*p* < 0.05). (G) Model-based Analysis of Genome-wide CRISPR/Cas9 Knockout (MAGeCK) analysis of the genome-scale screen showing top hits. (H) RNAi Gene Enrichment Ranking (RIGER) analysis of the genome-scale screen showing top hits. (I) Color-coded scatterplot illustrating hit identification for genes whose knockout suppresses tumor growth. The average sgRNA library representation in mBrie library transduced MC38 tumors (n = 6) was plotted against the average sgRNA representation in cell replicates (n = 4). The 15 top hits that pass all three statistical criteria were plotted as color-coded dots, where each dot representing an sgRNA with the same color indicating sgRNAs for the same gene. Non-targeting control sgRNAs (NTCs) were shown as dark gray dots, while other gene-targeting sgRNAs (GTSs) were shown as light gray dots. (J) Barplot depicting the number of independent scorings sgRNAs in the mBrie screen. (K) sgRNA performance by gene in cell and tumor samples, showing representative top 15 hits. In each gene’s plot, each different color represents a different sgRNA targeting the same gene.

**
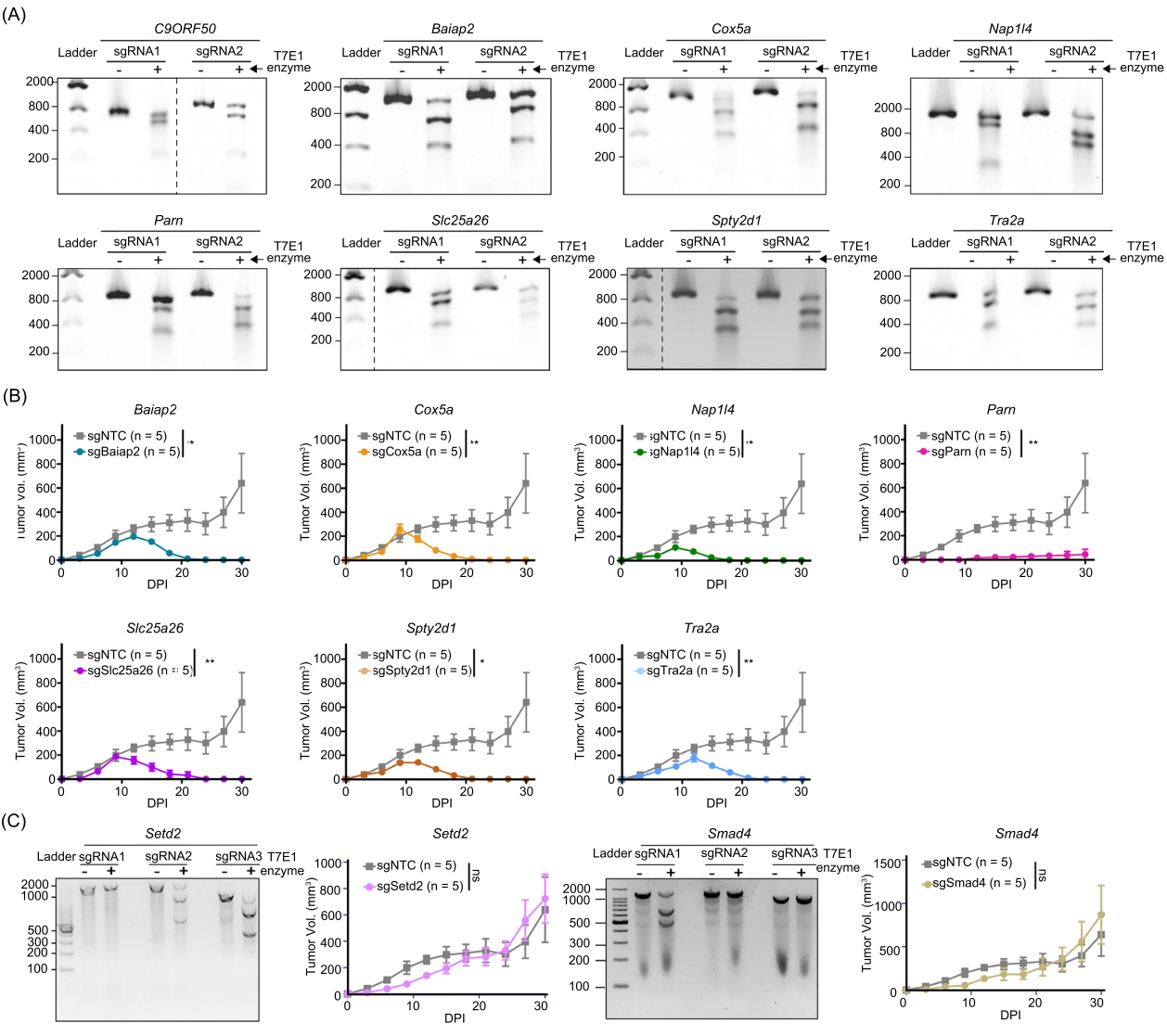
**

**Figure S2 Validation of the convergent top hits from genome-scale *in vivo* CRISPR screen.** (A) T7 Endonuclease I (T7E1) assay showing the knockout efficiencies of the sgRNAs for *C9ORF50, Baiap2, Cox5a, Nap1l4, Parn, Slc25a26, Spty2d1* and *Tra2a* (sgRNA1 and sgRNA2), examined 7 days after transduction. (B) Averaged tumor growth curves in mice of the *Baiap2, Cox5a, Nap1l4, Parn, Slc25a26, Spty2d1* and *Tra2a*. ***p* < 0.01 by two-way ANOVA with Holm-Sidak’s multiple comparisons test. (n = 5 per gene). (C) T7E1 assay showing the knockout efficiencies of the sgRNAs for well-studied tumor suppressor gene, *SetD2* and *Smad4* (sgRNA1 to sgRNA3), 7 days after transduction. Averaged tumor growth curves in mice of the *SetD2* and *Smad4*. No significant difference (n.s.) by two-way ANOVA with Holm-Sidak’s multiple comparisons test. (n = 5 per gene).


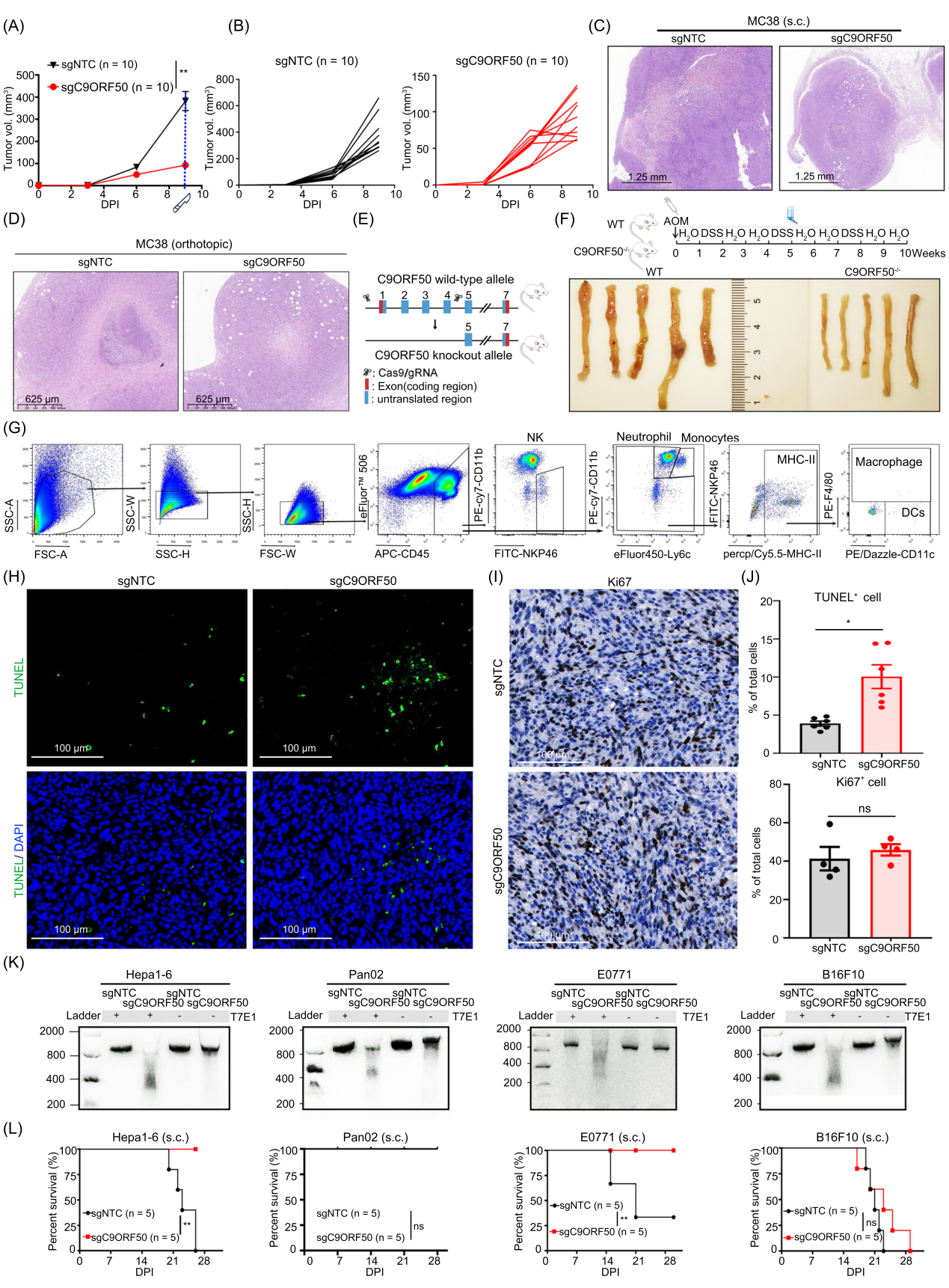


**Figure S3 *C9ORF50* knockout suppresses cancer progression.** (A and B) Tumor growth curves (A) and individual tumor growth curves (B) of transplanted MC38 cells transduced with sgC9ORF50 (red, n = 10 mice) or NTC (black, n = 10 mice) in C57BL/6 mice. 9 days after tumor transplantation, mice were sacrificed with the tumors collected and weighted. ***p* < 0.01 by two-way ANOVA with Holm-Sidak’s multiple comparisons test. (C and D) Histological observation of the heterotopic (C) and orthotopic (D) transplantation tumor tissues. The tumor sections were stained with H&E. Scale bar, 1.25 mm (C), 625 μm (D). (E) Schematic of the“Knockout-first” *C9ORF50* allele. (F) *C9ORF50* deficiency reduces inflammation-induced (AOM/DSS) colon carcinogenesis. Flow chart of the mice treated with AOM/DSS (above). Representative images of colon polyps induced by AOM/DSS in Wild-type (WT) and *C9ORF50*^−/−^ mice (bottom). (n = 5). (G) Flow cytometry gating strategy to identify different cell population. (H) Representative images of TUNEL (Green) IF staining in (C). Scale bars, 100 μm. (I) Representative images of Ki67 IHC staining in (C). Scale bars, 100 μm. (J) Quantification of Ki67⁺ and TUNEL⁺ cells were performed using ImageJ software. The percentage of Ki67⁺ cells was quantified from at least 1000 cells per mouse, with no significant difference (n.s.) observed between groups (Student’s *t* test; mean ± SEM; n = 4). In contrast, the percentage of TUNEL⁺ cells—also quantified from at least 1000 cells per mouse—showed a significant increase (**p* < 0.05; Student’s *t* test; mean ± SEM; n = 6). (K) T7E1 assay showing the efficiencies of the sgRNAs for *C9ORF50* (sgRNA1 and sgRNA2) 7 days after transduce to Hepa1-6, Pan02, E0771 and B16F10 cells. (L) Kaplan-Meier survival analysis showing the percentage of surviving of sgC9ORF50-transduced (red, n = 5 mice) or sgNTC-transduced Hepa1-6, Pan02, E0771 and B16F10 cells (black, n = 5 mice) transplanted mice. s.c., subcutaneous injection. The data represent the mean ± SEM, n = 3.


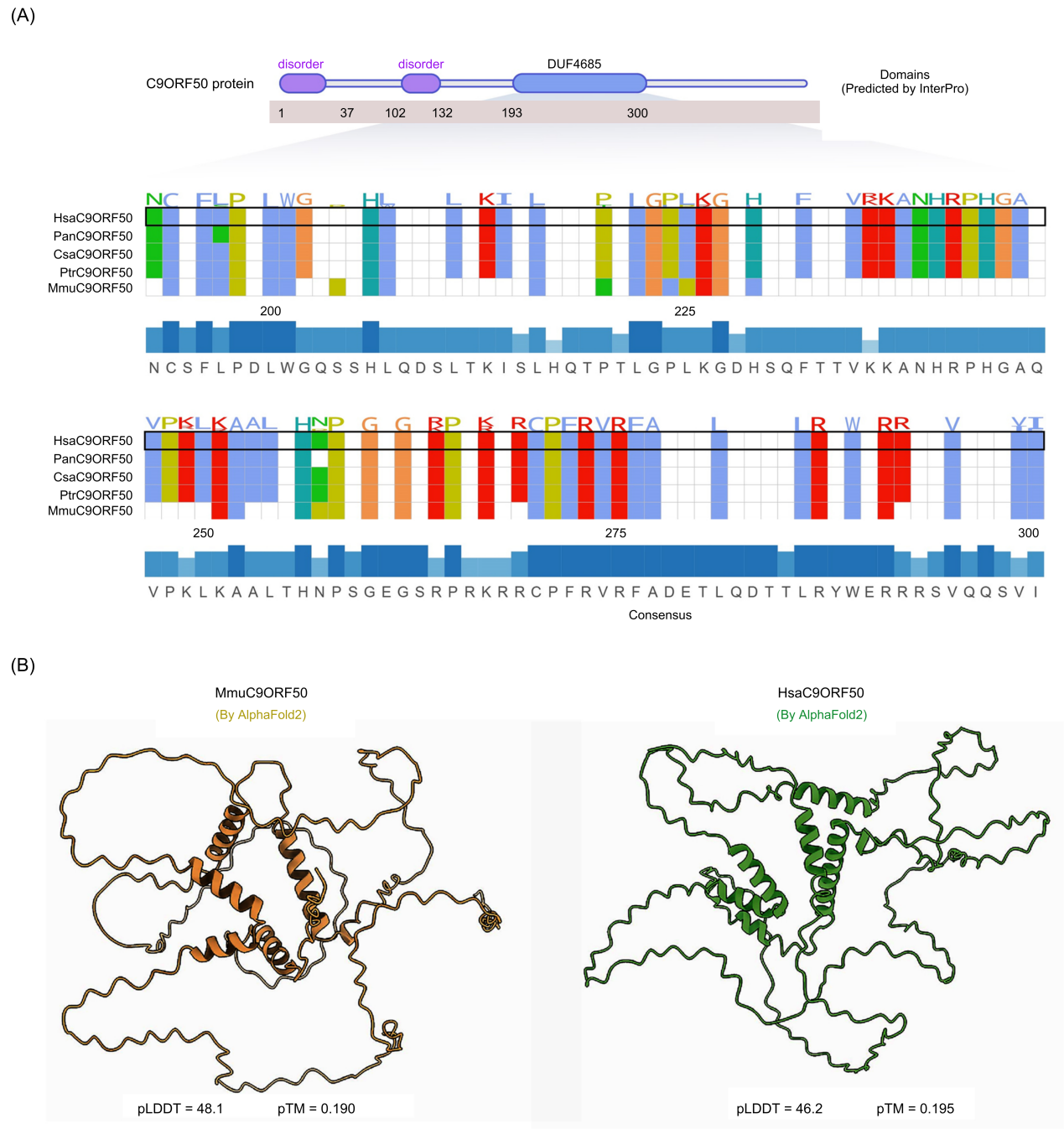


**Figure S4 Structural analysis of C9ORF50.** (A) Schematic showing the C9ORF50 protein secondary structure (at top) predicted using online tool (CFSSP). Multiple sequence alignment of various C9ORF50 proteins (at bottom). (B) Three-dimensional (3-D) structures of the C9ORF50 orthologue genes in human and mouse were predicted using AlphFold2.


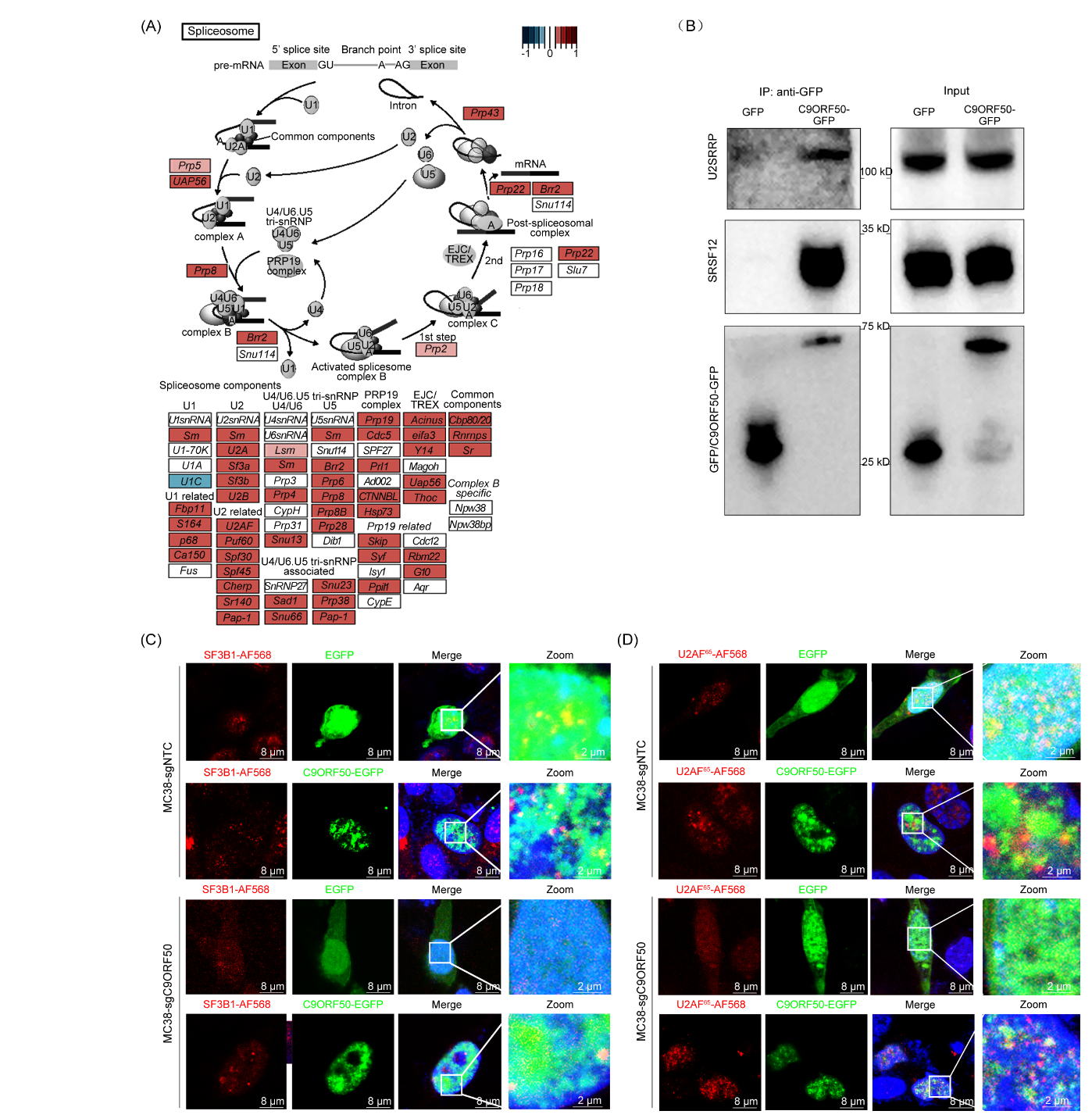


**Figure S5 C9ORF50 interacts with spliceosome components.** (A) The components of the spliceosome in the proteins interacting with the C9ORF50. (B) Western blot analysis confirms that both U2SURP and SRSF12 interacted with the C9ORF50. MC38 cells were transfected with pCMV-C9ORF50-GFP or pCMV-GFP plasmids. Cell lysates were immunoprecipitated with anti-GFP antibody, followed by immunoblotting analysis with antibodies against U2SURP and SRSF12. SDHA served as loading control. Images representative of 3 biological repeats. (C and D) Representative immunofluorescence of SF3B1 (C) and U2AF^65^ (D) in MC38 cells under four conditions: co-localization of GFP and SF3B1/U2AF^65^ in MC38-sgNTC cells; co-localization of C9ORF50-GFP and SF3B1/U2AF^65^ in MC38-sgNTC cells; co-localization of GFP and SF3B1/U2AF^65^ in MC38-sgC9ORF50 cells; co-localization of C9ORF50-GFP and SF3B1/U2AF^65^ in MC38-sgC9ORF50 cells. Column 4 displays 5× zoom of boxed regions of Merge. Scale bar, 8 μm or 2 μm. Images representative of 3 biological repeats.

**
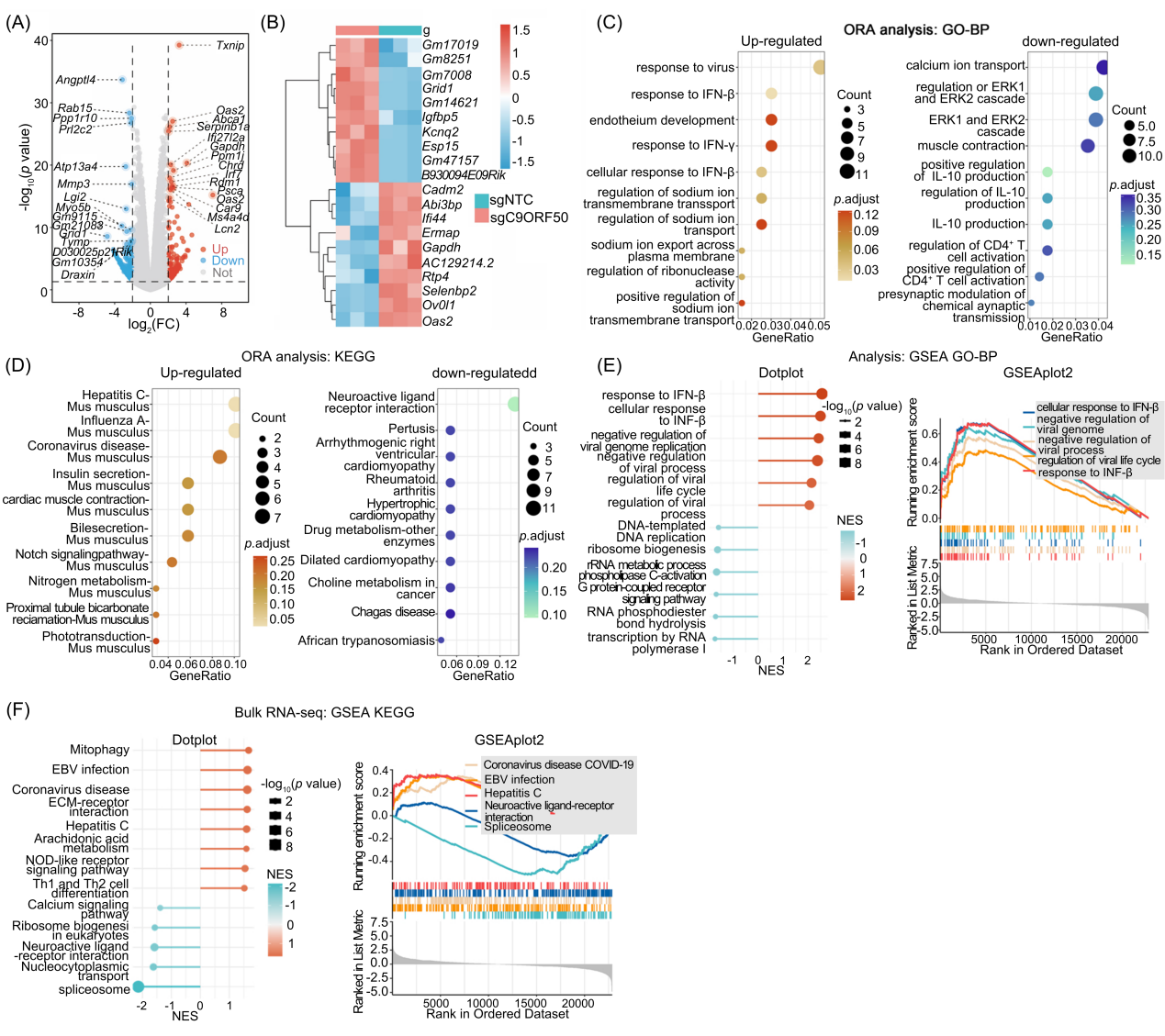
**

**Figure S6 Transcriptomic sequencing and data analysis.** (A) Volcano plots of differentiation gene expression in *C9ORF50* knockout MC38 cells compared with that in sgNTC MC38 cells. (B) Heat map depicting the significantly differentially expressed genes between indicated cells. Red indicates high relative expression, and blue indicates low relative expression. (C-F) The enriched gene sets in *C9ORF50* knockout MC38 cells compared with that in NTC MC38 cells are analyzed using ORA and GO-BP (C), ORA and KEGG (D), GSEA and GO-BP (E), GSEA and KEGG (F).

**
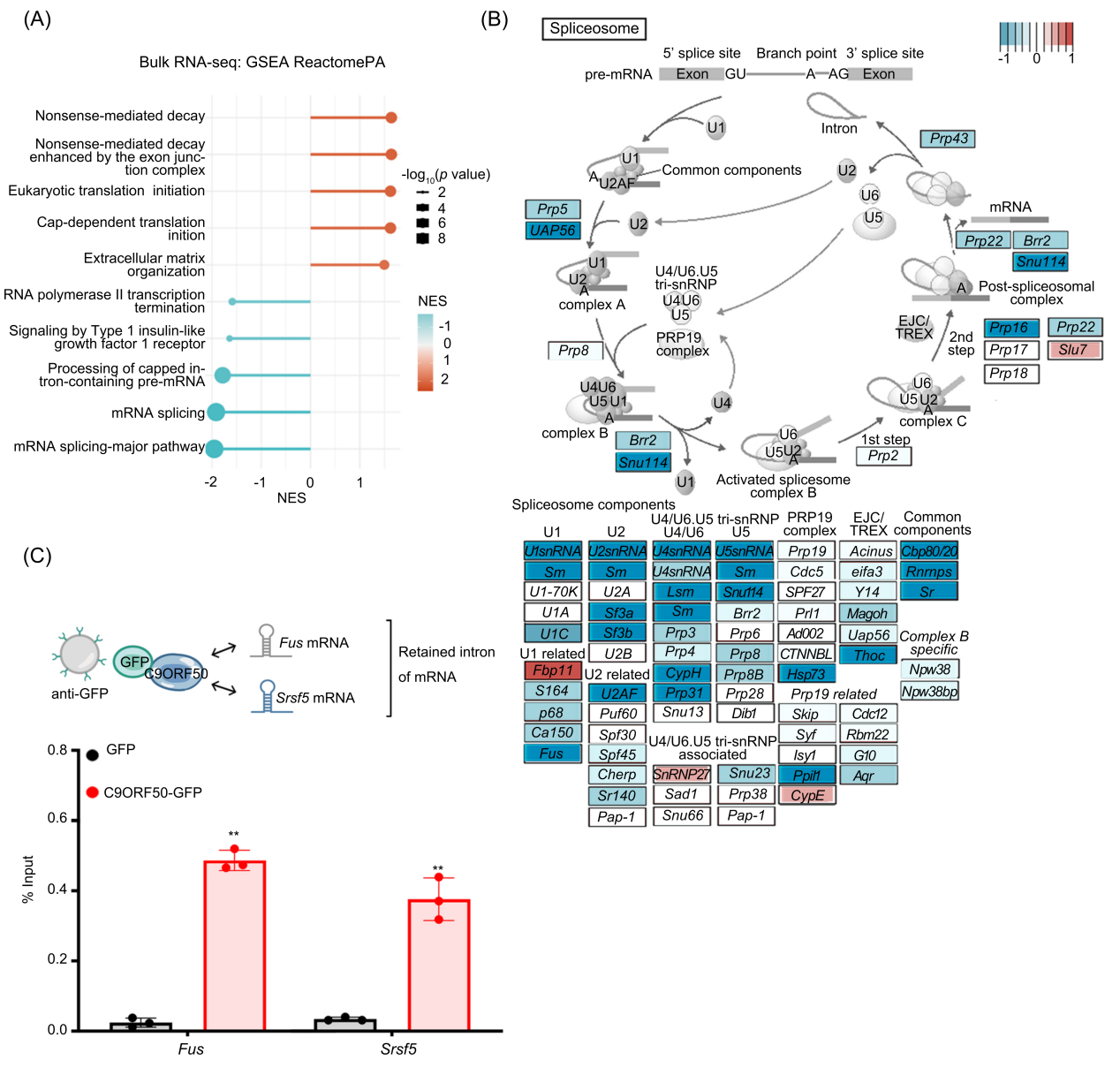
**

**Figure S7 Transcriptome analysis of RNA splicing process influenced by *C9ORF50* knockout.** (A) The enriched gene sets of RNA splicing regulation in *C9ORF50* knockout MC38 cells compared with that in NTC MC38 cells are analyzed using GSEA and Reactome. (B) The components of the spliceosome in the enriched genes in *C9ORF50* knockout versus NTC MC38 cells. Red, upregulated; Blue, downregulated. (C) Schematic diagram of the experimental design (above). RNA immunoprecipitation (RIP) with anti-GFP antibody followed by qPCR analysis of *Fus* and *Srsf5* mRNAs in MC38 cells transfected with C9ORF50-GFP or GFP control (bottom). ***p* < 0.01 by Student’s *t* test, Data are shown as mean ± SEM, n = 3.


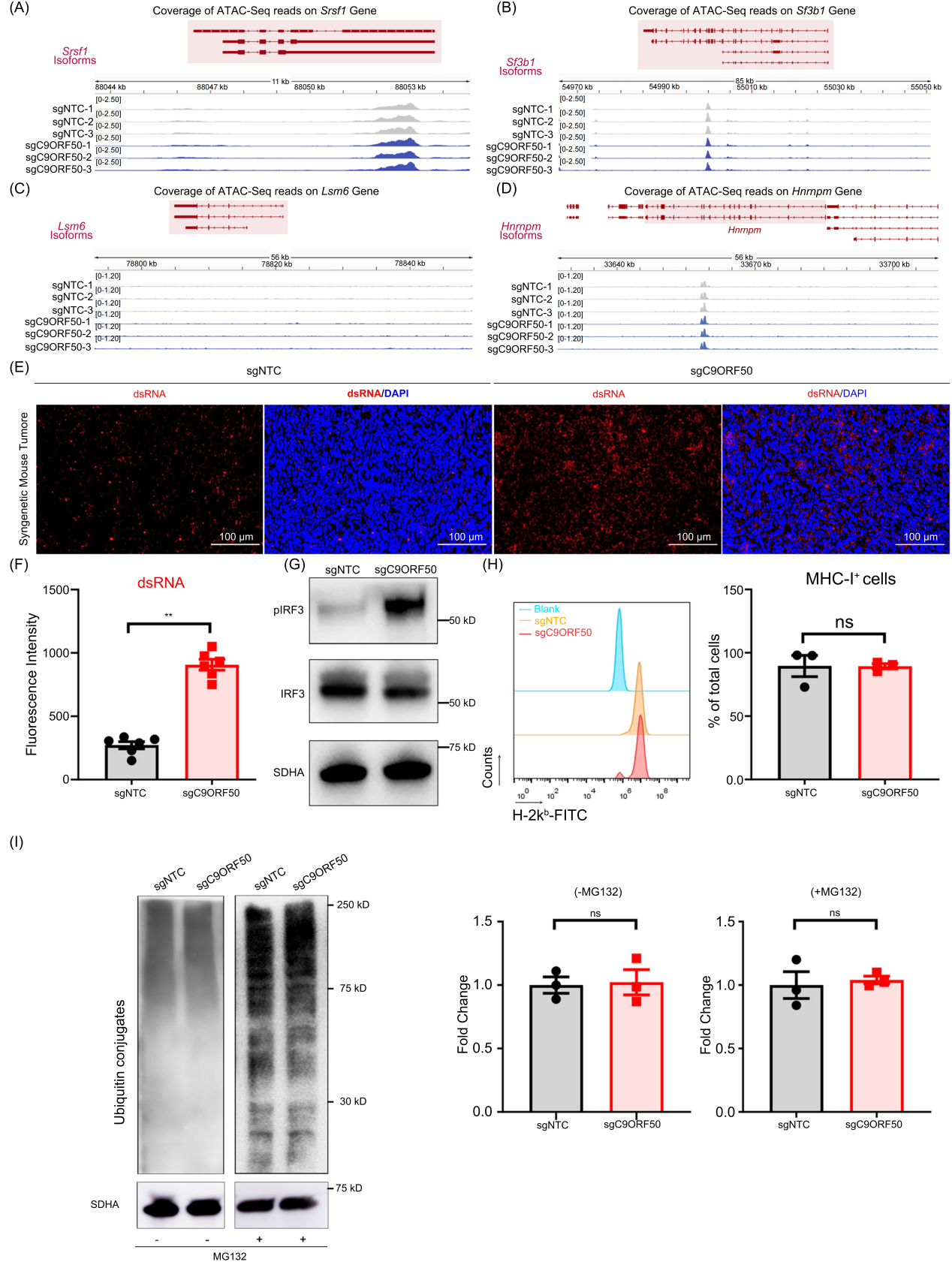


**Figure S8** ***C9ORF50* deficiency activates innate immunity through dsRNA sensing without affecting antigen presentation or global proteostasis.** (A-D) ATAC-Seq reads for representative spliceosome components influenced by *C9ORF50* knockout. (A) *Srsf1* gene. (B) *Sf3b1* gene. (C) *Lsm6* gene. (D) *Hnrnpm* gene. (E) Representative dsRNA immunofluorescence staining in the sgNTC or sgC9ORF50 tumors of mice. Quantification of dsRNA immunoreactivities. Scale bar, 100 μm. Images representative of 3 biological repeats. (F) Quantification of cytoplasmic dsRNA signal intensity by ImageJ software. ***p* < 0.01 by Student’s *t* test. Data are shown as mean ± SEM, n = 6. (G) *C9ORF50* deficiency leads to increase of the phosphorylation of IRF3 in *C9ORF50* knockout cells compared to in NTC cells. Protein levels of p-IRF3/IRF3 and SDHA were measured by Western blotting. SDHA served as loading control. Images representative of 3 biological repeats. (H) Flow cytometry analysis of total H-2K^b^ expression levels in the sgNTC or sgC9ORF50 cells. Quantification of H-2K^b+^ cells (MHC-I^+^) in the sgNTC or sgC9ORF50 cells. No significant difference (n.s.) by Student’s *t* test. Data are shown as mean ± SEM, n = 3. (I) Western blot analysis of ubiquitin (conjugated or free form) and SDHA in the sgNTC or sgC9ORF50 cells, with (+MG132) or without (−MG132) proteasome inhibitor treatment. Quantification of ubiquitin conjugates in the absence of proteasome inhibition, normalized to sgNTC. Images representative of 3 experiments. No significant difference (n.s.) by Student’s *t* test. Data are shown as mean ± SEM, n = 3.


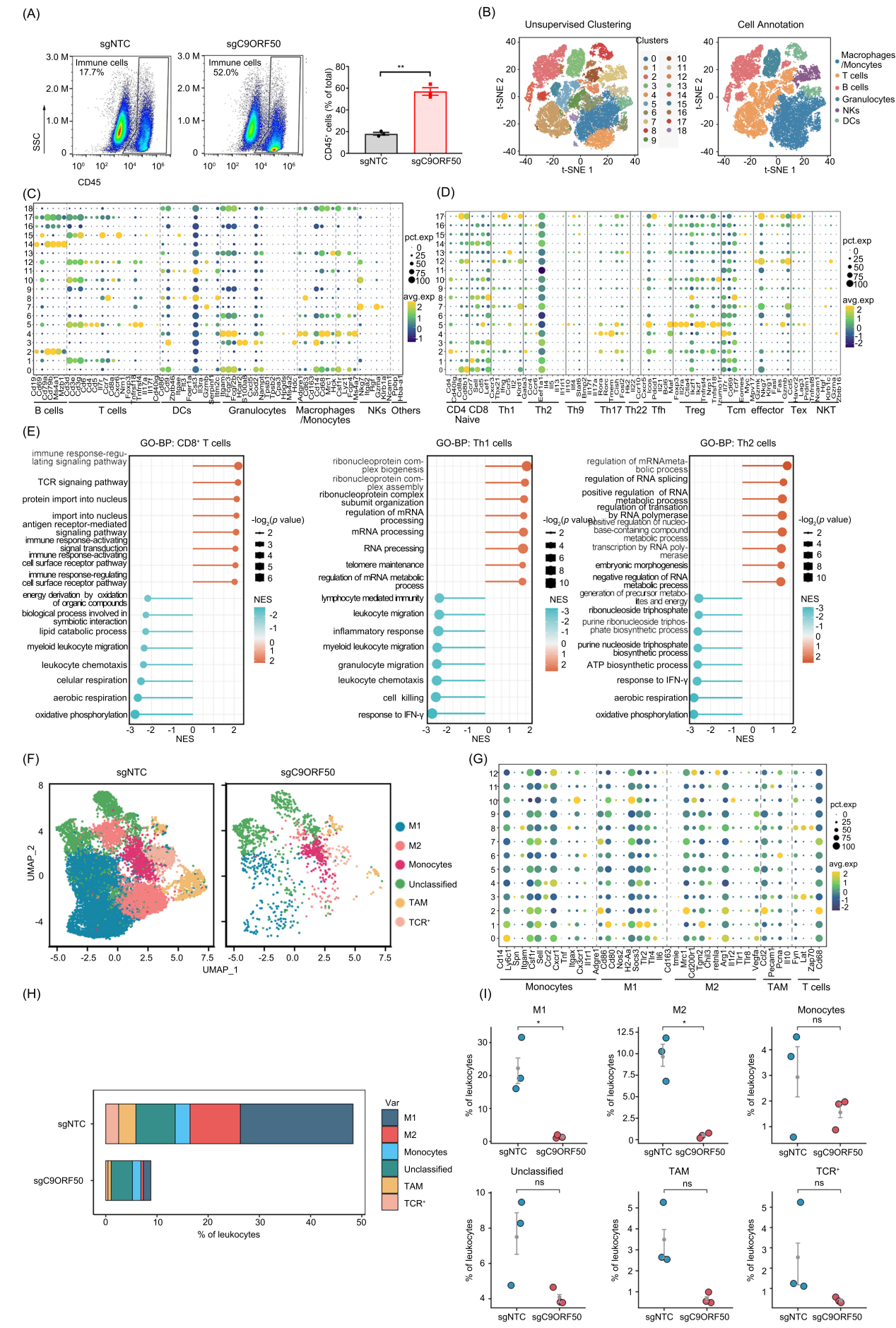


**Figure S9 Additional analysis of tumor immune infiltration profile influenced by *C9ORF50* knockout.** (A) Flow cytometry analysis of CD45^+^ immune cells within tumors. Representative images (left) and quantification analysis (right) of CD45^+^ leukocytes. The data represent the mean ± SEM, n = 3. ***p* < 0.01 by Student’s t test. (B) The tSNE plot showing the clustering of different cell subsets in *C9ORF50*-deletion-driven tumor (sgC9ORF50, n = 3 mice) or NTC tumor (sgNTC, n = 3 mice). 6 major leukocyte cell types including Macrophages/Monocytes, T cells, B cells, Granulocytes, NK cells, and Dendritic cells are shown. (C) Dot plot of 18 clusters with unique signature gene expression profiles of immune cells. (D) Dot plot of 17 clusters with unique signature gene expression profiles of T cells. (E) GO-BP analysis the DEGs in the CD8^+^ T, Th1, and Th2 cells revealed the enrichment of genes participating in the T cell receptor signaling pathway and immune response-regulating signaling pathway. (F) Uniform manifold approximation and projection (UMAP) plot re-clustering and re-annotating macrophage cells and other different cell subsets in the sgNTC or sgC9ORF50 groups. 6 major cell types are shown. (G) Graph showing the cluster proportions in the sgNTC or sgC9ORF50 groups. (H) Comparative analysis of macrophage cells and other different cell subsets infiltration between *C9ORF50*-deficient and control tumors. Bar plots quantify the relative proportions of tumor-infiltrating macrophage cells and other different cell subsets, including M1, M2, Monocytes, TAM, and unclassified cells. (I) Comparing the differentiation potential of cell subpopulations (M1, M2, Monocytes, TAM and unclassified cells) in the sgNTC or sgC9ORF50 groups. The dots and box lines show subgroup scores. *p* values were obtained using a two-sided Wilcoxon rank-sum (Mann–Whitney) test with Benjamini–Hochberg correction across subpopulations; **p* < 0.05, ***p* < 0.01. n = 3.


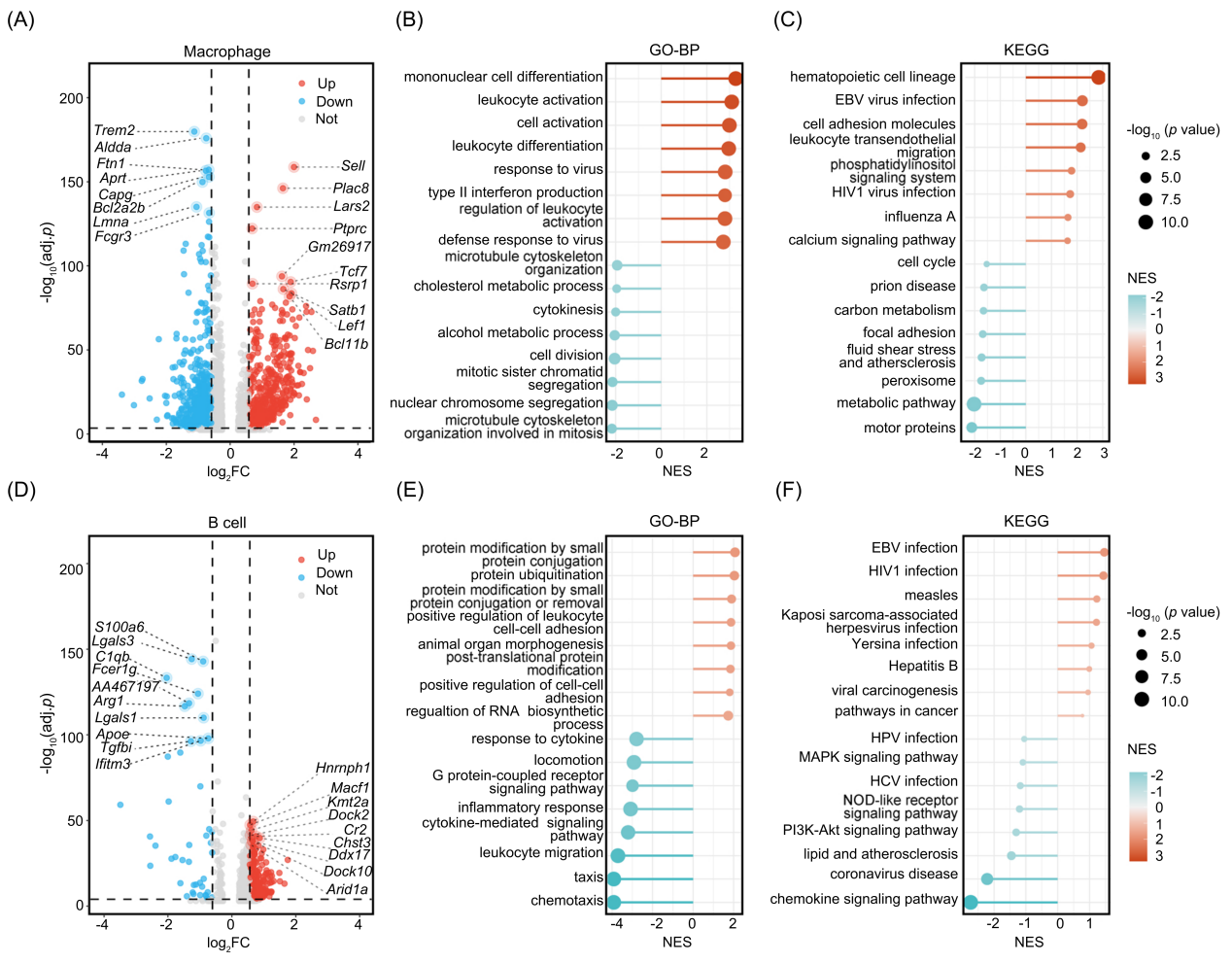


**Figure S10 *C9ORF50* knockout induces immune cell state reprogramming in the tumor microenvironment.** (A) Volcano plots of differentiation gene expression in macrophage from *C9ORF50*-deletion-driven tumor (sgC9ORF50, n = 3 mice) or NTC tumor (sgNTC, n = 3 mice). (B and C) GO-BP (B) and KEGG (C) analysis the DEGs in the macrophage cells. (D) Volcano plots of differentiation gene expression in B cells from *C9ORF50*-deletion-driven tumor (sgC9ORF50, n = 3 mice) or NTC tumor (sgNTC, n = 3 mice). (E and F) GO-BP (E) and KEGG (F) analysis the DEGs in the B cells.


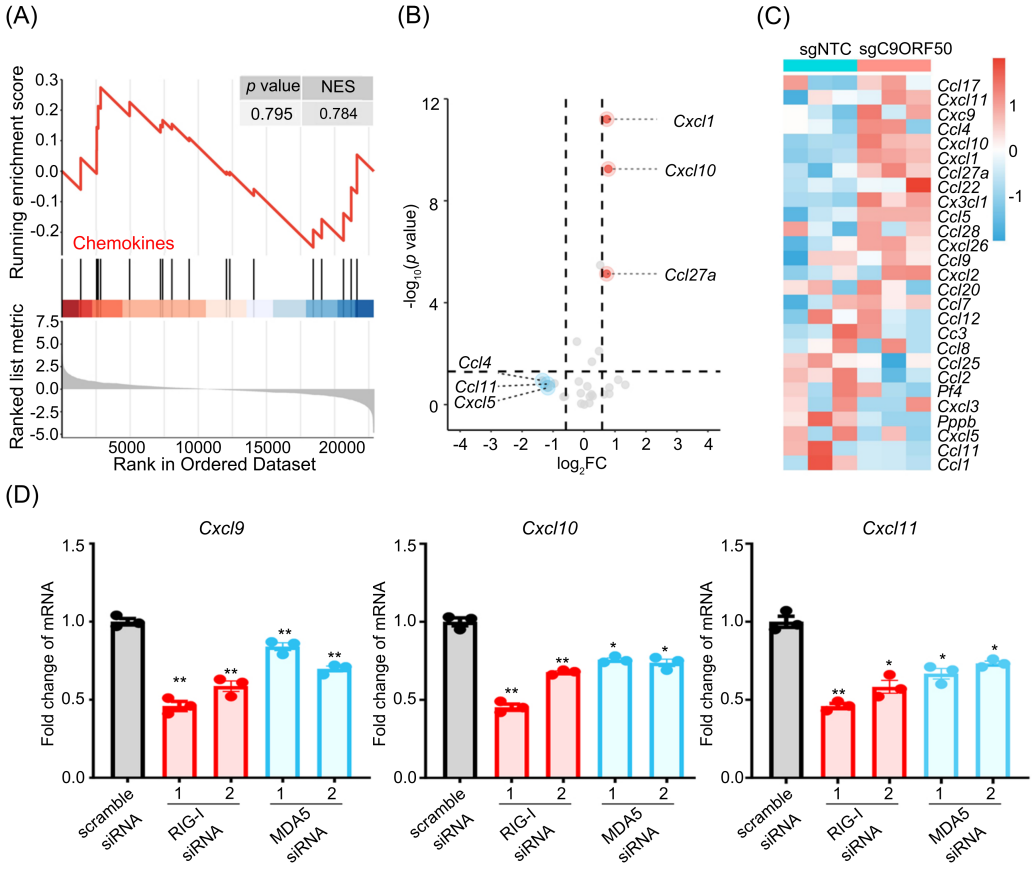


**Figure S11 *C9ORF50* deficiency leads to production of chemokines.** (A) The enriched gene sets of chemokines in *C9ORF50* knockout MC38 cells compared with that in NTC MC38 cells are analyzed using GSEA. (B) Volcano plot of chemokines in *C9ORF50* knockout MC38 cells compared with that in NTC MC38 cells. (C) Heatmap of the chemokines in *C9ORF50* knockout MC38 cells and NTC MC38 cells. (D) *RIG-I* and *MDA5* contribute to expression of chemokines in *C9ORF50* knockout MC38 cells. MC38-sgC9ORF50 cells were transfected with control siRNA (Scramble siRNA) or siRNAs targeting *RIG-I* and *MDA5*, and assessed for *Cxcl9, Cxcl10* and *Cxcl11*. **p* < 0.05, ***p* < 0.01 by Student’s *t* test. Data are shown as mean ± SEM, n = 3.


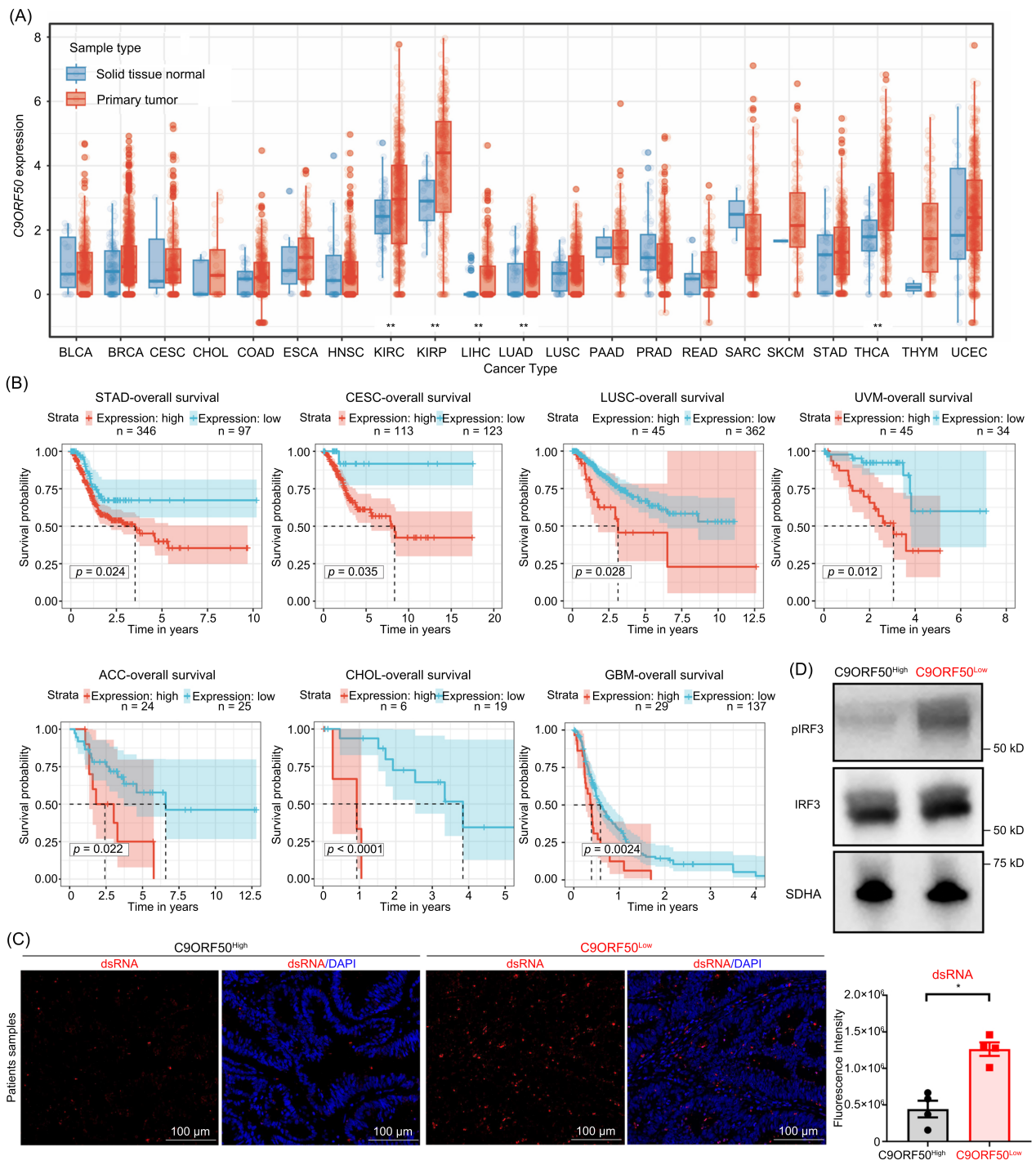


**Figure S12 *C9ORF50* deficiency correlates with enhanced anti-tumor immunity and improved survival in colorectal cancer.** (A) Differences of *C9ORF50* gene signatures between tumor and para-carcinoma tissues. ***p* < 0.01 by two-way ANOVA. (B) Kaplan–Meier overall survival analysis of Stomach Adenocarcinoma (STAD), Cervical Squamous Cell Carcinoma and Endocervical Adenocarcinoma (CESC), Lung Squamous Cell Carcinoma (LUSC), Uveal Melanoma (UVM), Adrenocortical Carcinoma (ACC), Cholangiocarcinoma (CHOL) and Glioblastoma Multiforme (GBM), based on the *C9ORF50* expression. The *p* value was obtained by log-rank test. (C) Representative dsRNA immunofluorescence staining in *C9ORF50* expressed high or low human colorectal cancer tumors samples. Quantification of dsRNA immunoreactivities. Quantification of cytoplasmic dsRNA signal intensity by ImageJ software. Scale bar, 100 μm. Images representative of 3 experiments. **p* < 0.05 by Student’s *t* test. Data are shown as mean ± SEM, n = 6. (D) *C9ORF50* deficiency leads to increase of the phosphorylation of IRF3 in *C9ORF50* expressed low human colorectal cancer tumors samples compared to high. Protein levels of p-IRF3/IRF3 and SDHA were measured by Western blotting. SDHA served as loading control.


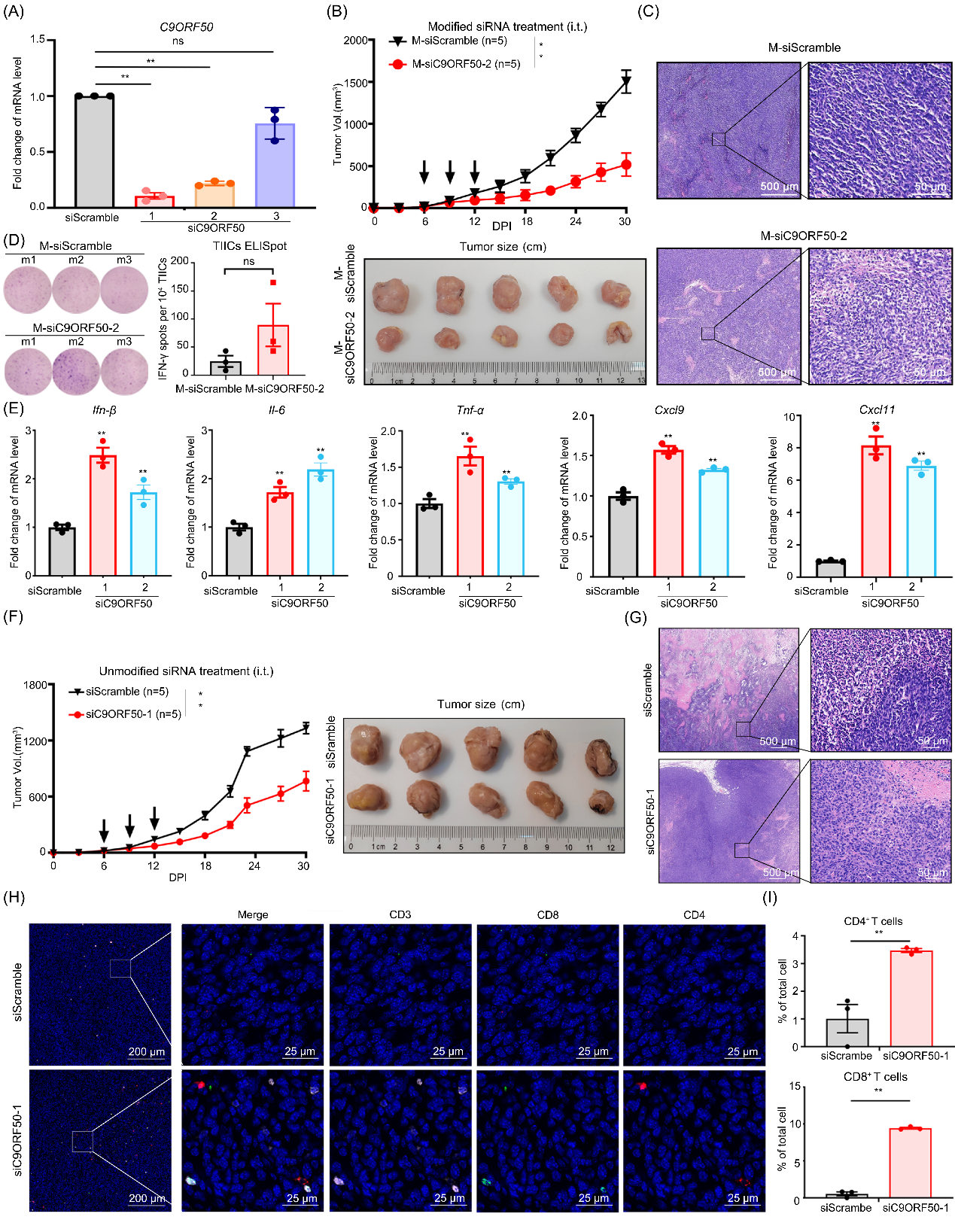


**Figure S13 Additional analysis of cancer therapeutic effect of RNA interference targeting *C9ORF50*.** (A) qPCR analysis of *C9ORF50* expression 24 h after *C9ORF50* siRNAs transfection. Three C9ORF50-specific siRNAs (siC9ORF50-1/-2/-3) were designed and tested separately compared with negative scrambled siRNA (siScramble) set to Control. No significant difference (n.s.) (p > 0.05), ***p* < 0.01 by Student’s *t* test. Data are shown as mean ± SEM, n = 3. (B) Tumor growth curves after intratumoral injection (i.t.) of cholesterol-C9ORF50-siRNA-2 (M-siC9ORF50-2, n = 5 mice) or cholesterol-scramble-siRNA (M-siScramble, n = 5 mice). ***p* < 0.01 by two-way ANOVA with Holm-Sidak’s multiple comparisons test. Photographs of tumors derived from each treatment group. (C) H&E-stained tumor slices after different treatments. Scale bar, 500 μm, 50 μm. Images representative of 3 biological repeats. (D) Representative images (left) and quantification (right) of IFN-γ ELISpots performed on tumor-infiltrating immune cells (TIICs) isolated from M-siC9ORF50-2 or M-si-scramble tumors. No significant difference (n.s.) (p > 0.05) by Student’s *t* test. Data are shown as mean ± SEM, n = 3. (E) Knockdown of *C9ORF50* with siC9ORF50-1 or siC9ORF50-2 significantly upregulated mRNA expression of *Ifn-β*, *Il-6, Tnf-α*, *Cxcl9*, and *Cxcl11* in MC38 cells. The data represent the mean ± SEM, n = 3. ***p* < 0.01 by Student's *t* test. (F) Tumor growth curves after intratumorally injection (i.t.) of siC9ORF50-1 (n = 5 mice) or siScramble (n = 5 mice). **p* < 0.05 by two-way ANOVA with Holm-Sidak’s multiple comparisons test. Photographs of tumors derived from each treatment group. (G) H&E-stained tumor slices after different treatments. Scale bar, 500 μm, 50 μm. Images representative of 3 experiments. (H) Representative CD3, CD4 and CD8 multicolor immunofluorescence staining in siC9ORF50 or siScramble tumors. Scale bar, 25 μm. Images representative of 3 experiments. (I) Quantification of CD3, CD4 and CD8 immunoreactivities. The data represent the mean ± SEM, n = 3. ***p* < 0.01 by Student’s *t* test. Data are shown as mean ± SEM, n = 3.
